# Dictionary of Practical Medicine

**Published:** 1847-07-01

**Authors:** 


I. A Dictionary of Practical Medicine. By *James Copland,* M.D., F.R.S., &c. &c. Parts X. and XI. Article—Hæmagastric Pestilence (Yellow Fever).

II. Report of a Special Committee of the House of Assembly of the State of New York, on the present Quarantine Laws. Albany, 1846.

III. Report of the Fever at Boa Vista. By Dr. *Mc William,* R.N. Presented to the House of Commons, in pursuance of their Address of the 16th March, 1847.

IV. Letter addressed by Sir William Pym to the Lords of the Council, relative to a Report on the Fever at Boa Vista by Dr. McWilliam. Presented to the House of Commons, in pursuance of their Address of May 14th 1847.

In recent Numbers of this Journal we have considered at some length two of those wide-spread and devastating diseases, to which the term of Pestilence has been more peculiarly applied by Dr. Copland, and which he has treated of apart and by themselves in his great work on Practical Medicine. The especial object of our remarks in these articles has been to examine the important question—important in a political and commercial, as well as in a strictly professional, point of view—whether the diffusion of the diseases in question over large districts of the earth can with reason be attributed to their direct transmission from one individual to another, or whether it be not mainly owing to some peculiar, although unknown and almost inscrutable, condition of the atmosphere, over which the utmost efforts of man can have no controul, save and except by removing all those causes of insalubrity which, it is well known, invariably add malignancy to morbific miasms, while at the same time they render the human body not only more susceptible of their influence, but also less capable of resisting their fatal operation. In the case of the Plague, abundant cause was shewn that many of the opinions, that have hitherto been maintained (for the last century, at least), are utterly erroneous, and therefore that the system of Quarantine that has been based upon these opinions has been most absurdly vexatious, and most unnecessarily oppressive. The great relaxations of the restraints to commercial intercourse, that have been made within the last six months by the Governments of England and France, are the gratifying results of our more accurate and enlightened knowledge of the disease;—thanks chiefly to the admirable Report of the French Academy, with which our readers are so well acquainted.

With respect to the Epidemic Cholera, the evidence adduced in our last Number will be deemed sufficient, we trust, to satisfy almost every impartial mind that infection, whether by persons or by the medium of inanimate objects, was a very subordinate and merely an occasional agent in the diffusion of that extraordinary pestilence, which almost girdled the earth in its career, from the wall of China on the East to the banks of the Ohio and the St. Lawrence on the West. Its history is especially interesting to the medical philosopher, as presenting, on the whole, by far the best instance on record where we have a full and complete account of the rise, progress, and gradual and progressive extension of a great and destructive epidemic. For a period of twenty years, it appeared to be travelling on from one part of the world's surface to another. Not that, during this time, it always followed a direct or an uninterrupted course along any one tract; for we know that this was not the case. Still, its general march was onward and advancing from East to West; as, indeed, has been observed to be the case with some other wide-spread diseases. And never were the attempts of man to stay the progress of an epidemic distemper more signally—we might almost say ludicrously—impotent. Truly it may be said that, in the event of a second irruption of the Cholera into Europe, it would be as reasonable to expect to screen a province from the invasion of a pestiferous blight by building a lofty wall around its borders, as seek to keep out that disease from a country by any system of quarantine establishment. And here we cannot but suggest how very useful an accurate history of certain blights and mildews—and what are they but pestilences of the vegetable world?—might be to the elucidation of that of epidemic disorders. Is it not probable that there is something more than a mere analogy between their exciting causes? Nay, is it unlikely that their origin is sometimes the very same? At all events, the comparative investigation of animal and vegetable pestilences affords, we humbly think, the fairest prospect of our ever attaining to anything like a reasonable hypothesis as to the cause or causes which give them birth. The appearance of the potatoe blight among us has invested the subject at this present time with more than ordinary interest, and may thus have the effect of awakening the attention of scientific men in general to the vast importance of studying the wide-spread diseases of plants.* But this only in the way of parenthesis.

We shall now proceed to the more immediate subject of the present article; viz. the history of the Yellow Fever, or, as Dr. Copland proposes to call it, the Hæmagastric pestilence,† more especially in reference to its probable causes, its relations with other diseases, the general mode of its propagation, and the means to be employed to prevent its development or arrest its extension. The subject, it may be remarked

---

* Has any accurate account of the chronological and geographical history of the potatoe disease been published? Where and when did it first make its appearance? Have different countries been cotemporaneously or successively visited by its invasion? How long has it remained in each country, and what are the circumstances of soil, locality, &c. that seemed to favour or resist its incursions? Although Mr. Smee and some other writers have collected a good deal of information elucidatory of these questions, there is no complete history of this singular pestilence: at least we know not of any.

† From αιμα *blood,* and γαστηρ *the belly;* so called, we presume, from the black sanguineous discharges, upwards and downwards, from the alimentary canal.

*in limine*, is in some respects surrounded with much greater difficulties than attend the like history of its two predecessors; difficulties that chiefly arise from the circumstance that medical writers, even in the present day, are far from being agreed among themselves as to what assemblage of symptoms or morbid phenomena the appellation of Yellow Fever is to be restricted. A somewhat similar difficulty, indeed, occurred in the case of the Epidemic Cholera; for Dr. Copland, it will be remembered, has endeavoured to establish a specific distinction between that disease and the endemic cholera, that had been long known in the East. But the grounds adduced for this distinction we found to be altogether so very insufficient, that it seems not at all likely that many will be inclined to adopt the same opinion. With respect, however, to the Yellow Fever, the point is by no means so easily disposed of; and indeed the solution of this very point is, as we have remarked, the chief difficulty that awaits us in the following enquiry.

All our readers are probably aware that, for the last half-century and upwards, a very keen and occasionally very acrimonious controversy has been going on among medical men, more especially among those belonging to the naval and military services, as to the true nature of the disease so designated. One party contends that more than one malady or morbid state, although dissimilar (it is asserted) in phenomena as well as in origin and attributes, have been most inaccurately confounded together under a single name, and that it is to this unfortunate confusion of things really and essentially distinct, that all the perplexities of the subject are owing. The gentlemen who hold this opinion assert that Yellow Fever is a disease *sui generis*, and as specifically different from all other forms of fever as Small-pox, Scarlatina, or Measles; and they maintain that it has no alliance whatsoever, save only what is merely coincident and accidental, with any other malady of the country where it happens to appear. On the other hand, a large proportion of writers on the diseases of hot climates regard Yellow fever as merely an aggravated and malignant form of the ordinary endemic fever of these climates; the only difference between them, according to these gentlemen, being in degree and not in kind. We frankly confess that much may be said, and said, too, with force and shew of reason, on both sides of the question, and that its solution is far from being so very easy, as many writers on both sides of the controversy have far too dogmatically asserted.

The appearance of the last two parts of Dr. Copland's Dictionary of Practical Medicine appears to us to afford a singularly favourable opportunity to sift and try the merits of the question fairly. Our reasons for saying so are these. The objection that has always been made by the one party against the other, by the advocates of the specific and essentially distinct nature of the Yellow Fever against those who will not admit this doctrine, is that their opponents have confounded different diseases together, and have thus too often ascribed to one the characters and attributes which really belong only to the other. If, for example, an appeal be ever made by the latter to the writings of Bancroft and Jackson, for evidence or authority upon one of the disputed points in the history of the disease, they are at once met with the reply that these writers never distinguished with any degree of accuracy the genuine pestilential disease from other fevers, which, although resembling it in many respects, are really and truly of a very different nature. Now it is not easy, it must be confessed, to get rid of this objection; resolving itself, as it does, into a simple assertion which it is often impossible, from the absence of sufficient, or at least admissible, data, to gainsay. To attempt to settle the dispute by a mere counter-assertion will never do; this is only entangling and perplexing the subject the more. An angry controversy arises; the most conflicting and contradictory statements are made; facts are distorted to serve a particular purpose; and the disputants become, it is generally too obvious, more anxious about the triumph of their own opinions than the discovery and elucidation of the simple truth. It cannot be denied that, among medical writers, controversial discussions are rarely carried on with that spirit of courtesy and firmness which might be reasonably expected from men engaged in so honourable a profession as ours.

But to return to our line of argument: we were alluding to the objection that will assuredly be urged against any appeal to the works of many of the most able and experienced writers upon the subject. Now, how is this objection to be obviated? Not certainly by balancing the numerical strength of the two contending parties, nor yet by calculating the comparative ability or means of observation of the opposing authorities, nor even by judging from the alleged results of their experience in different climates and in all variety of circumstances. We must therefore follow another plan, and try if we can steer clear of the difficulty that has been mentioned. The argument we shall follow is this. If indeed Bancroft, Jackson, and other writers on the same side have erred in the way supposed, the same thing cannot surely be said of Dr. Copland, who has not only written at great length on the various diseases involved in the controversy, but has also laboured with the utmost energy to establish those very distinctions that have been so stoutly denied by the gentlemen now named. That his own party will be willing to accept his championship of their cause, we cannot for a moment doubt; for where can they find an advocate more zealous for their cause, and better, or indeed so well, prepared to enforce their arguments, and repel the attacks of their adversaries? His immense erudition, his intimate acquaintance with all that has been advanced on the subject, his great experience in marshalling his facts, so that they may appear as strong and conclusive as they possibly can do, and in exposing the weakness or fallacy of those which do not favour his views, are all well known, and will naturally cause him to be looked up to as the most learned and able expositor of his side of the question. Such then being the case, it only remains to be seen whether his opponents are equally ready to admit his descriptions, as the standard by which to test the soundness of those principles which he labours to inculcate. In the present article we purpose to do so; and, by comparing together the accounts which he has himself given of those diseases that are (it is asserted) so liable to be mistaken for each other, to endeavour to arrive at something like a high degree of probability—we do not presume to speak of certainty in this matter—upon the point at issue, viz. the nature of Yellow Fever; whether, in short, it is a disease specifically and essentially distinct from every other, or whether it is but a modified form of the common endemic fever or fevers of the country where it happens to prevail.

It may be as well to prefix the general definition which Dr. Copland has given of the malady. It runs thus:—*After chills, shivering, languor, severe pain in the orbits and forehead, also in the loins and limbs;—rapid pulse, flushed face, glassy suffused eyes;—peculiar burning heat of skin and frequent delirium—nausea and vomiting with epigastric pain, costiveness, great anxiety, restlessness, and watchfulness; subsequently hiccough, black vomiting, scanty or suppressed urine, hæmorrhages from the mucous canals, lemon or muddy yellowness of skin, generally terminating in death in its most severe form.*

The yellow discolouration of the skin and the black vomit are by some writers considered to be characteristic or pathognomonic phenomena of the disease; but Dr. Copland admits that neither of these symptoms is uniformly or invariably present, and moreover that both the one and the other occasionally occur in other descriptions of fever. "The term *yellow* fever," he says in one passage, "ought to be entirely discarded; as yellowness, being improperly viewed as a pathognomonic symptom of one kind of fever, all others, in which it is a contingent phenomenon, although not more frequently met with in one than in another, have been confounded with that fever." After alluding to the different shades of the discolouration of the surface, he adds:—"It may proceed either from the passage of the colouring parts of the bile into the circulation, or from incipient dissolution of the blood, with loss of the vital tone of the capillaries."

With respect to the *black vomit*, it is admitted, in more passages than one, that it is not necessarily present even in the worst and most rapidly fatal cases of the disease. If such then be the case with the two most conspicuous and remarkable symptoms, we cannot be surprised that the others, enumerated in the definition, are still more uncertain and fallacious. Truly has Dr. Gillkrest, one of the most recent and candid writers on the subject,* remarked that "the anomalies which this disease has been observed to present—the absence, under the observation of one medical man, of some of the symptoms which during another epidemic had been well marked—the fact of practitioners having observed that certain symptoms, prominent during one period of an epidemic, have at another period been totally absent—the fact that of patients in the very same ward of an hospital being frequently found to labour under symptoms so variously grouped as to lead an inexperienced practitioner to believe that he had before him three or four diseases bearing little affinity to each other;—all these circumstances have thrown difficulties in the way of this disease having had a place assigned to it in nosological arrangements free from objections." Of the perfect truth of these observations the reader will soon be convinced, by the perusal of the details which we now intend to bring under his notice.

And first with respect to the very close resemblance that may be traced between the different degrees or modifications of the genuine pestilence, and some of the ordinary endemic fevers of intertropical regions. This point we shall illustrate by presenting in opposite columns the descriptions of each, as given by our author himself.

---

* Cyclopædia of Practical Medicine. Vol. II., p. 264.

*Yellow Fever.*—"The mildest form of this pestilence generally makes its appearance with languor and slight chills, soon followed by heat of skin; quick and full pulse; uneasiness in the loins and limbs; severe headache, confined chiefly to the orbits and forehead; a peculiar shining or drunken appearance of the eyes; hot dry skin; a loaded but moist tongue, with little thirst; sickness at stomach, with costiveness, and a feeling of uneasiness, not amounting to pain, at the epigastrium, and a sense of rawness or soreness in the fauces and in the course of the œsophagus. These symptoms may continue from twelve to twenty-four or thirty-six hours, when the patient, having taken only some purgative and febrifuge medicines, or an emetic, falls into a refreshing sleep; from which he awakes in a gentle perspiration, free from pain and fever, and complaining only of debility, from which he rapidly recovers." Vol. III., p. 139.

*Bilio-gastric Fever.**—After the usual premonitory symptoms of a febrile attack, there "soon succeed severe frontal headache, vertigo, nausea, vomiting, burning heat of skin, restlessness, watchfulness, slight anxiety at the præcordia, pain and oppression in the epigastrium, and in one or both hypochondria, with more or less soreness, fulness, and tenderness. The eyes are moist and injected, the conjunctiva often yellowish; the face is flushed; the breathing oppressed and accelerated; the pulse full, large, quick, and strong, rarely hard; the tongue is clammy, moist, furred, and yellowish, with a bitter taste in the mouth; the thirst is urgent, the breath fætid; the bowels are obstinately costive, or loose; the stools bilious, and the urine scanty and dark. When the stomach and bowels are inordinately affected, cerebral congestion very frequently supervenes at a later period. As the disease advances, the pulse feels less full, and is weaker than in health. The thirst and anxiety are increased; and the upper parts of the body are sometimes covered by a profuse sweat whilst the skin still continues hot." Vol. I., p. 984.

That there may be no mistake respecting the Bilio-gastric Fever here spoken of, it will be well to give the following few particulars touching its history :—

"This fever is either sporadic, endemic, or epidemic.—It is *endemic* in warm countries and marshy situations among Europeans, particularly those who have not been long resident in these parts : and in marshy localities in the summer and autumn in temperate climates.—It is *epidemic* in some seasons, particularly in autumn when the summer has been hot, after a wet spring, or after great falls of rain, or after inundations, and when great numbers of predisposed persons, especially from high latitudes, visit such localities. In these circumstances and persons, it proves the seasoning fever. It is observed chiefly in adults of the bilious or bilio-sanguine temperaments, and in persons addicted to spirituous liquors. It is a very prevalent fever in the countries bordering on the Mediterranean, in the East Indies, and in America, and consequently in fleets and armies in these parts.

"Gastro-bilious fever is *caused* chiefly by exhalations from the soil, or from vegetable and animal matter undergoing decomposition, in connection with at-

---

* The fever, so denominated by our author, is the common "bilious," or "gastro-bilious" fever of many writers. It is very well defined by Dr. Copland thus :—*Vascular re-action following chills or rigors and other symptoms of premonition and invasion, with predominant affection of the biliary functions, and of the digestive mucous surface; frequently with yellowness of the skin in the severer cases.*

mospheric heat; by exposure to the sun; by the night airs or dews, and the influence of cold following such exposures or excessive exertion or high ranges of temperature; by intemperance and errors of diet or of regimen; by excesses in vinous or spirituous liquors." P. 984.

With regard to the *mild form* of the true Yellow Fever, all that requires to be said respecting it is that, however slight and unalarming the symptoms may be, the disease is declared by our author to be essentially and invariably infectious; so much so that, in his opinion, the most fatal and malignant infection may originate from a case of the least possible severity. He acknowledges, indeed, that in this mild form it cannot be distinguished but with very great difficulty from the common bilious or bilio-gastric fever; so nearly do their symptoms resemble each other. This difficulty, too, must be not a little enhanced by the circumstance, admitted by Dr. C., that the latter may acquire, under peculiar circumstances, infectious properties. "The influence of infection in producing it," says he, "has been doubted; but the experience of Drs. Denmark and Boyd, in ships and hospitals in the Mediterranean, has demonstrated its occasional origin in the cause—or, at least, the power infection evinces in producing a severe modification of it."

Before taking leave of the two kinds of fever described above—viz. the Bilio-gastric and the mild form of Yellow-fever—we must not omit to state that there is quite as close an analogy or alliance between the Remittent fever of warm climates and these, as there is between the two kinds themselves. This alliance is rendered the more conspicuous from the fact admitted by our author, that the bilio-gastric fever not only arises from the same causes as the remittent, but also not unfrequently lapses into the periodic type. Compare the following description of the first form or degree of the latter with those already given:

*Mild Remittent.*—"The stage of *invasion* is similar to that already described; it being attended by coldness of the surface, and frequently by shivering. The coldness is soon superseded by heat, by febrile flushes, or by alternations of heat and cold, by nausea, and occasionally by vomiting, which soon develop the stage of *excitement.* With it, the pains of the head, back, and limbs become remarkably aggravated; the mouth is clammy and dry; the tongue white or loaded; the surface very hot and parched; the face flushed; the features tumid; and the pain of the head attended by a feeling of distension and throbbing, often passing into delirium. The pulse, which, at the invasion, was small, irregular, and weak, is now full, large, strong, and frequent; thirst is urgent; the bowels constipated; and the urine scanty and high-coloured. There is always more or less tenderness at the epigastrium, with nausea, and often with vomiting. These symptoms generally continue from about ten or twelve to eighteen hours, when perspiration breaks out; the pulse falls in frequency and strength; the irritability of the stomach subsides; delirium disappears; and the skin becomes cooler: but there is merely a remission or abatement, but no intermission of the febrile symptoms. The remission usually continues from three to nine or ten hours, when an exacerbation occurs, sometimes preceded by chills or shiverings, at other times not, and the severer symptoms are renewed. Thus the disease proceeds with alternate remissions and exacerbations, the former generally taking place in the morning, until the seventh day, or the ninth, eleventh, or fourteenth day, or much later, in temperate countries, when a copious perspiration generally puts a termination to its progress." Vol. I., p. 947.

Having thus shown how nearly the milder form of the true pestilential fever of the West Indies and of the African coast resembles, in its outward features at least, the common endemic fevers, remittent as well as continued, of these countries, we shall now advance a step farther in our *comparative* investigation of their histories, and this we shall do according to the same plan we have followed above.

*Yellow Fever.*—" The more severe and more frequent form appears more suddenly (than the mild form already described—*Rev.*) and the symptoms are much more violent. The attack is ushered in by shivering and rigors. The pain in the orbits and forehead is excruciating; severe pain is also complained of in the loins and calves of the legs; the face is flushed; the eyes are glassy, suffused, or apparently inflamed; the pulse is rapid; the skin burning hot and dry; and the tongue is loaded, but moist, with little thirst. A few hours afterwards, uneasiness of stomach, with nausea and vomiting, supervenes; followed by severe pain and tenderness at the epigastrium, with a sense of rawness, heat, or inflammation in the fauces and down the œsophagus; great anxiety, restlessness, and watching, with a desire of sleep. The bowels are constipated, the evacuations scanty and deficient in bile: the urine dark-coloured and small in quantity.

" If the disease be judiciously treated, these symptoms often become ameliorated on the second or third day; the patient falling into a sleep, from which he awakes refreshed, with a perspiring or moist skin, and nearly free from all the symptoms. Debility only remains, the recovery from which is generally rapid. In many cases, however, either a partial amelioration only occurs, or the more complete subsidence of the symptoms is of short duration; the patient in a few hours beginning to be troubled with flatus in the stomach, and distressing hiccough. Not unfrequently the patient is suddenly and unexpectedly seized with faintness, sickness, and painful retchings, followed by vomiting, at first of whatever had been taken into the stomach, but soon afterwards of a

*The Ardent or Seasoning Fever.**— " The attack is usually sudden. Giddiness, faintness, and general uneasiness, sometimes, however, precede it for ten or twelve hours. There is, occasionally, a slight and brief chilliness at the commencement, especially in the less violent cases, rapidly followed by a sense of universal heat; by flushed face, frontal headache, and vertigo; by inflamed, heavy eyes, and great sensibility to light and sound; by pain in the occiput, neck, back, and limbs; and by a strong, full, hard, and accelerated pulse. A sense of heat, oppression, pain, or anxiety, is felt at the præcordia, sometimes with a dry cough, and pain in the side; respiration is quick, laborious, suspirous, or anxious; the tongue is white, excited, and its edges red; the fauces are arid, thirst urgent, and skin hot and dry; the urine is scanty, the bowels costive; and there is generally nausea, but seldom vomiting until some time after the attack. If the disease be not mitigated by treatment, the patient becomes extremely restless; the headache is rending and intense; vascular action is excessive; and the heat very great. Vomiting now supervenes, and follows the ingestion of whatever is taken to allay the urgency of thirst. The matters thrown off are generally tinged with bile; and a bilious yellow suffusion of the skin is frequently observed. Bilious vomiting and purging occasionally occur with the yellowness of the surface, and, in the slighter cases, become a favourable crisis. There is often great drowsiness, but no refreshing sleep. These symptoms of excessive excitement proceed with various degrees of violence, and occupy a period of from twenty-four to sixty hours, but most commonly from twenty-four to

* This is the *Endemial Causus* of Moseley, the *Inflammatory Endemic* of Dickenson, and the *Endemic Yellow Fever* of some writers.

brownish fluid, resembling dirty water, mixed with a dark-coloured flaky matter, which floats upon its surface; and at last, by a matter resembling coffee-grounds or thin pitch. At this time, also, a great change takes place in the countenance, which assumes a putrid, dingy, and bloated appearance, which is most remarkable in those of a florid or sanguine complexion. A light yellow or lemon tinge appears under the eyes and ears, and soon spreads down the neck to the chest, and over the whole body. The vessels of the conjunctiva appear relaxed, and distended with blood. The vomitings continue, and the quantity of fluid ejected much exceeds that which has been drunk. They often return without being excited by ingesta; or even suddenly or unexpectedly, and when the patient has just before considered himself relieved from them."

" The *third form* of attack also commences with shivering or rigors, and is an aggravation of the symptoms of the second from the beginning. In this form, the face is more flushed, and the burning heat of skin is greater than in the preceding. The sickness of stomach, hiccough, and black-vomiting appear much earlier. The bowels are obstinately constipated, and resist strong purgatives; the motions being watery, of a dirty colour, and rarely feculent or bilious. Violent delirium often occurs early in the attack, and hæmorrhages frequently take place at an early period from the nose, mouth, eyes, ears, and even from all the outlets of mucous canals. The tongue is often clean, moist, livid, or red, and raw-like, or covered with dissolved blood. The action of the kidneys is suppressed, either little or no urine being secreted. The countenance changes to a livid and yellowish hue, with yellowness of the skin. In the most severe of these attacks the patients may be carried off on the second, but generally on the third day, sometimes in convulsions." P. 139.

" The *blood* is always more or less changed—most remarkably after the calm occurring on the third day. Even at the commencement and during re-action, it does not coagulate, or does so forty-eight hours. During this period, blood taken from a vein is remarkably florid, warm, and fluid. The fibrin coagulates firmly, but the crassamentum is without crust, and is rarely cupped.

" The excitement having reached its acme, is quickly followed by exhaustion, This is indicated by a subsidence of the most urgent symptoms: the pain and heat are lessened; the skin becomes damp or clammy; and the patient has a sense of cold or slight chilliness. This delusive remission is a state of great danger: in some cases, it passes into rapid sinking — into a speedily fatal collapse."

" *Discolouration* of the skin generally takes place in this stage; appearing in yellow, yellowish brown, and livid patches. It never occurs in the period of excitement, for it is quite dissimilar from the bilious yellowness occasionally observed in that period. It is commonly attended by passive hæmorrhage from the nose, gums, eyes, ears, &c., and by black and grumous vomiting. The change of colour, and hæmorrhage, proceed from exhaustion of the vital influence in the extreme vessels, and from the changes induced in the mass of blood. The matters thrown off the stomach consist at first of ingesta and serous fluid, often coloured by bile. In a more advanced stage they are ropy, mixed with numerous small shreds, flocculi, or films, which soon acquire a dark brown, purple, or black colour; but do not, at first, communicate much of the same tint to the fluid containing them. Afterwards, the matters vomited are more intimately mixed; and, from dark-coloured blood which has been effused into the stomach, vitiated bile, and other morbid secretions, assume a dark or coffee-grounds appearance. At the same time, dark-coloured matter, resembling tar mixed with black blood, is freely discharged from the bowels."

" The *blood* at this period is black, thin, and dissolved, its fibrin seems diminished, and it does not separate into crassamentum or serum; or if it does, the former consists of a thin dark jelly, with the black colouring matter precipitated towards the bottom of the vessel." Vol. I., p. 977.

imperfectly and loosely, and is deficient in fibrine. It afterwards becomes still more loose and defective as to crasis, and ultimately very dark, partially dissolved, and grumous; and apparently insufficient in quantity, in many cases, to distend the veins." Vol. III., p. 141.

Dr. Copland remarks that the Ardent or Seasoning Fever, " after the inflammatory excitement is subdued by copious depletions, sometimes assumes a *remittent* character."* He also expressly says that, " it is *not liable to recur.*" " A first attack," he adds, " prevents a second, if the individual continue in the climate which caused it; but, if he return to a cold country, and reside there until the energy of his system is restored, he becomes liable, upon his return to the hot climate, to a second attack, although less so than before, and in a milder form." He afterwards qualifies this statement by saying that a second attack is rare even in persons who may have returned to a temperate climate and then gone back to their tropical residence; the seasoning fever, under these circumstances, being much more frequently of a *remittent* than of a *continued* type. The Seasoning Fever, we are also told, "will not prevent those diseases which proceed from marsh exhalations; but, if the person who has been seasoned by it, be seized with fever from this cause, the periodic type will be assumed." Lastly, Dr. C. does " not believe that this—the climate or seasoning-fever—will exempt from the pestilential yellow-fever, although it may lessen the susceptibility to it, when the individual has not intermediately changed the climate. Instances are numerous of seasoned persons—of those who have suffered from this, the climate or severe inflammatory, fever—afterwards being seized with endemic or remittent fever, or with the pestilential disease (genuine yellow fever)."

This form of fever " is the disease which most frequently attacks new comers into the West Indies, more especially sailors and soldiers. * * * * * * * It was also very prevalent during the last war among the British troops and sailors in the Mediterranean, and was described by Burnett, Irvine, Boyle, Brunton, and others; but it generally assumed a milder form than in the West Indies." Dr. C. does not allude to this fever ever exhibiting *infectious* properties, unless perhaps the fact implied in the following remark:—" If animal or vegetable miasms concur with them (the usually exciting causes, viz. a very high temperature, often conjoined with rich, nutritious and heating food, stimulating drinks and suppressed perspiration), the fever will present adynamic or malignant characters in proportion to the activity of either of these agents." Here, before proceeding further, a good opportunity occurs of alluding to the nature and cause of the " black vomit" and black discharges from the bowels, that constitute so remarkable a character of certain fevers in hot climates. The following excellent remarks on the subject are introduced by our author at the close of his description of the Climate or Seasoning fever.

---

* " Mr. Martin, speaking of the ardent fever of Bengal, says—" The type of this fever is usually *continued,*—but sometimes *remittent.*"—*Influence of Tropical Climates, 6th Edition.*

"The source of the black matter passed from the stomach and bowels in the last stage of this and of other severe fevers of warm countries, has been variously stated. Some consider the black colour to proceed from the exudation of dark blood, which, in mixing with the secretions of the stomach, liver, and bowels, imparts to them a still darker tint. Some ascribe it chiefly to the bile, and secretions from the digestive mucous follicles, which are often both very dark and thick, in the last stage of the more malignant kinds of intertropical fevers; and others believe it to arise both ways. There is no doubt that all the secretions poured into the digestive canal are more or less diseased, particularly in the latter stages: but it is as clear, that the black colour mainly depends upon the state of the blood; and that all the matter ejected upwards and downwards, presenting this appearance, does not consist of altered secretions merely,—a great part of it probably being an exudation of blood from the mucous surface. I believe, also, that these matters vary very remarkably in the ardent climate fever, in the more malignant forms of marsh or endemic fevers, and in the pestilential yellow fever —the diseases thus characterised. Dr. Jackson remarks that the secretions from the digestive mucous surface are ropy and clear during the early periods, and are brown or black in the latter—sometimes black as soot; and that the sooty or ink-like colour is chiefly observed where the head and stomach are simultaneously attacked. When we consider that the blood becomes darker than natural, as well as otherwise changed, early in the period of exhaustion, and that the liver and mucous follicles of the digestive canal, with the kidneys, are the principal organs of depuration, or channels by which the elements producing these changes are eliminated from the circulation, we need not be surprised at the secretions, which these elements go to form, and which these organs excrete, presenting somewhat similar characters. It must however be admitted, that the share which the secretions perform in producing this phenomenon, or that which the exudation of blood has in giving rise to it, will vary much in different varieties or cases of intertropical fevers.—The rapidity with which a dissolution of the tissues takes place after death, in the severe forms of climate fever, deserves notice, as marking the rapidity of vital exhaustion, and as resulting from the changes of the blood; these changes commencing with the stage of exhaustion, and advancing until this fluid is no longer capable of influencing the nervous system, and of preserving the irritability of contractile parts—or until it poisons, instead of exciting, the sensitive and moving tissues." Vol. I., p. 980.

We may here observe that it is scarcely, if at all, possible to perceive any distinction between the *ardent* or climate fever mentioned above, and the *inflammatory* and the *bilio-inflammatory* forms of *remittent* fever, as described by our author. Every symptom, that occurs in the one, is met with in the others, and in exactly the same progression. They are seen under the same circumstances, in the same regions, and are most frequent in the same class of persons, viz. in Europeans recently arrived in hot climates. The only (apparently) recognisable distinction between them is, that the one is spoken of as a continued, whereas the other is described as a remittent, fever. But then this distinction will not always hold, according to our author's own admission. We have already seen that the ardent fever sometimes puts on a remittent type; and now we find that the inflammatory and the bilio-inflammatory forms of remittent fever, are apt at times to become continued. For example, of the former we read that, "if the disease be neglected at the beginning, the *remissions disappear*, the skin becomes dry and caustic, or moist and clammy; the pulse small and irregular; the tongue black and crusted; and the vomiting, pain at the epigastrium, &c., more constant. In the most severe and unfavourable cases, *yellowishness of the skin, or vomitings of matters like coffee-grounds, or both,* occasionally supervene. * * * If the disease be not actively treated at the commencement, an unfavourable termination takes place between the 3rd and 7th day ; but it is often prolonged beyond this period, and it then generally occasions visceral disease."

Nearly the same characters are assigned to bad cases of the "bilio-inflammatory form" of the disease : " *the countenance and skin become dusky or yellow ;*" and, " after vomiting has continued some time, the appearance of the matters is changed, and ultimately assumes, in fatal cases, the characters just described ;" *i. e.* like coffee-grounds.

We need scarcely say that it must be extremely difficult to distinguish such cases of aggravated Remittent (or semi-remittent) from the genuine Yellow fever, more especially as it (the former) also, like the bad form of the Ardent or seasoning fever, chiefly affects new comers into tropical countries, and occasionally prevails there epidemically in unhealthy seasons, as stated by Dr. Copland.

But there is yet another form of endemic tropical fever described by him as the *adynamic* or *malignant remittent,* which brings us a step nearer still to the very worst cases of the genuine Hæmagastric pestilence. We shall now give his description of the former and the remaining portion of the description of the latter (the earlier portion having already been given in page 190), that the reader may judge of the close resemblance between these " essentially distinct diseases," and also between them and the malignant form of ardent fever, already described.

*Yellow Fever.*—"In plethoric persons and in the sanguine temperament, the attack is often most violent; and in addition to the symptoms just mentioned, the countenance appears bloated and heavy, with an unnatural expression, or wild and agitated. The heat of the surface, which was at first great and pungent, falls first in the extremities, and afterwards over the whole body, especially after the occurrence of black vomiting; and ultimately it sinks below the natural standard. The skin becomes compacted, losing its vascularity, and is insensible to the irritation of blisters. It is rarely dotted with petechiæ, but much oftener streaked with yellowish lines, particularly in the course of large blood-vessels, or is covered by patches of a blueish or leaden colour, especially in flaccid parts. The sense of internal distress increases as the febrile action subsides. Distension of the hypochondria, and explosions of flatus from the stomach, are frequent, with occasional obscure hiccuppings. Sometimes the vomitings are hardly complained of until the more febrile symptoms begin to

*Malignant Remittent.* — " In some cases the vascular excitement is at first more or less intense, with remarkable determination to the head, liver, and stomach, and maniacal delirium, the disease very nearly approaching the inflammatory, or bilio-inflammatory forms. In others, vascular reaction is very low and imperfect; the pulse small and quick ; the abdomen tumid and hot, whilst the extremities are cold or clammy ; the evacuations foul, morbid, and offensive ; the tongue fuliginous ; the gums spongy, or oozing a bloody sanies; the vomiting constant, and ultimately grumous and dark ; the stools, towards the close, black or pitchy; the urine scanty or nearly suppressed ; the solids flaccid; and the skin earthy and discoloured. In both these states, a yellowness of the surface occasionally presents itself about the third or fourth day, beginning in the conjunctiva, neck, and breast. The yellowness often passes to a pale greenish hue, in patches, shortly before death; and the soft solids present a liquescent state, having lost their vital cohesion.

abate, when they become unrestrainable: the matters ejected are then muddy or turbid, like unstrained coffee; occasionally they are of inky blackness, like the juice of the cuttle-fish. The evacuations by stool sometimes also present a black appearance at this stage. In the more severe states, the disease frequently terminates fatally within the fifth day. In the less severe cases, signs of an imperfect crisis sometimes appear about the seventh day; and improve to favourable indications; but occasionally they are arrested in their course, and superseded by an unfavourable train of symptoms, as hæmorrhages from the throat, gums, mouth, and sometimes from other outlets of mucous canals. The blood is dissolved, dark, incoagulable or grumous, particularly at a far advanced period of the disease.

"The *fourth form* of the pestilence seems a modification of the symptoms by temperament and habit of body, although the precise conditions of these cannot always be assigned, the phlegmatic, apparently, most frequently exhibiting it. In this the symptoms are not so violent as in the third form, but they are equally fatal. It often commences insidiously, the patient complaining, for hours, or even longer, of nothing but languor or fatigue, which is followed by chilliness or rigor, with pains in the loins and calves of the legs. The headache is not very severe. The pulse is quick and small. The heat of skin is very little increased; but there are great anxiety and oppression at the precordia, and an indifference to surrounding objects. The bowels are obstinately confined, and the secretion of urine is arrested. The tongue is often unnaturally clean, and of a clear shining vermilion colour. Hæmorrhage appears early from the nose, gums, or mouth, and is sometimes attended by petechiæ and vibices. There is little or no thirst, but great irritability of stomach, with hiccough and black vomiting, attended sometimes, as the distemper proceeds, especially towards the fatal close, by an involuntary discharge of the same ap-appearance from the bowels. The peculiar change of countenance, with yellow skin, takes place as in the other

"In other cases of this form, the symptoms are at first mild, and the excitement inconsiderable; when, after two, three, or four exacerbations, the powers of life appear suddenly exhausted; the pulse becomes weak and fluttering; the tongue foul, black, and dry; the evacuations offensive; the prostration of strength extreme; and the fætor of the perspiration remarkable. At last, great anxiety; tenderness and tension of the epigastrium; fulness of the hypochondria; collapsed features; a squalid or yellowish surface; vomiting of dark or grumous matters, supervene, and indicate the utmost danger. This insidious modification of the adynamic form generally occurs in persons highly predisposed, or who have suffered from bowel complaints, or who are debilitated and are subjected to the more concentrated effluvia.

"In some instances the remittent commences in so mild a form, that the patient is even able to walk about his apartment; and, for several days, complains only of irregular exacerbations of fever, when, suddenly, violent and malignant febrile action supervenes, which rapidly exhausts vital power, and either quickly carries off the patient, or induces serious structural change in several of the abdominal organs. In other cases, vascular excitement is hardly manifest at any period of the disease, the exacerbations consisting merely of increased anxiety, restlessness, general distress, and mental depression, occasionally with augmented sickness; and pain in the head, epigastrium, and loins; the pulse being but little accelerated until the close, and the temperature, unless at the epigastrium, rather under than above natural. In these, however, the weak, soft and open or irregular pulse; the dark-coated, or soft, flabby, and lobulated tongue; and the blackish, greenish-brown, and morbid excretions, in connexion with the other symptoms, denote extreme danger. It would seem as if the causes had nearly annihilated the irritability of the moving fibre, and deprived the system of its ability of re-acting upon or superseding, the morbid condition induced by their first impression."

states, and is frequently accompanied with a low muttering delirium."

"In the most violent seizures, the patient is suddenly struck, and the distemper proceeds rapidly without re-action, or nervous or vascular excitement, to vital and structural dissolution, with every indication of extreme vital depression, of vascular contamination, and of impaired or nearly lost irritability and cohesion of the tissues." * * * "When the infectious agent is very powerful relatively to the constitutional powers of the patient, the attack may then be so violent, and its subsequent course so malignant, as to deprive the vital energy of all power of reaction. In this case, the invasion is sudden and severe, and is attended with general tremor, dread, terror, or despondency; the vital depression of this period passing into vital and even structural dissolution, with greater or less rapidity, and either with no attempts at reaction, or with weak and abortive efforts merely." In the majority of cases, however, the stage of vital prostration is preceded by one of high and violent febrile excitement. Vol. III., p. 140 & 142.

To this minute description we shall merely add, that this malignant form of intertropical remittent "is variously *modified*, in different circumstances and persons. It sometimes assumes more of a *cerebral or typhoid character;* at others, it is *bilious or gastric*, according to peculiarity of season or concentration of the cause. In some intertropical countries it becomes epidemic, or rather this endemic is more than usually prevalent. Occasionally the remissions are indistinct from the commencement, and they generally become so after three or four days." Vol. I., p. 948.

As affording a graphic illustration of a *malignant remittent* in a tropical region, we shall here insert the account of an epidemic fever at Batavia, as given by Mr. Shields, and inserted in Dr. Johnson's work on Tropical Climates. We are the more desirous of drawing the reader's attention to this narrative, from the circumstance of certain writers having inaccurately stated that the fevers of the East never exhibit the peculiar features—we allude to the discolouration of the skin and the black vomit—of those of the West Indies and of the African coast, and too dogmatically asserted that the true yellow fever is wholly unknown in the eastern hemisphere.*

Edam, where the dreadful pestilence was experienced, is a small island, a few miles distant from Java: it is over-run with jungle, and is full of marshes. The time of the year was August, when the sun was nearly vertical. After some introductory remarks, Mr. Shields observes:

"The patient, without much previous notice, was suddenly seized with giddiness and cold chills, sense of debility and vomiting, and with pain over the orbits and in the epigastric region. He frequently fell down, and was insensible during the paroxysm; his body covered with cold clammy sweats, except at the pit of the stomach, which always felt hot to the palm of the hand: the pulse was small and quick. On recovering a little, the train of symptoms was succeeded by flushings of heat, increased pain over the orbits and in the sinciput, pain and a

---

* It is rather singular that one of the earliest appellations of the yellow fever was *Maladie de Siam.*

sense of internal heat about the stomach and præcordia, oppressed breathing; the lower extremities at this time not unfrequently covered with cold sweats. The eyes now become as it were protruded, and the countenance flushed. Retching, and at length vomiting of discoloured bilious matter came on; the tongue was white and furred, and the abdomen tense and full, with pain in the loins and lower extremities. The length of this paroxysm varied from 6 to 18 hours, and was generally succeeded by cold rigors; very often low delirium, preparatory to the next stage or paroxysm of the fever. The intellectual faculties now became much impaired, the patient not being at all sensible of his situation or of any particular ailment. If asked how he was, he generally answered 'very well,' and seemed surprised at the question. This was a very dangerous symptom, few recovering in whom it appeared. In this stage all the symptoms became gradually often rapidly aggravated; particularly the headache, pain and tension in the epigastric region and vomiting. Some patients on shore were carried off in 18, 24, 30 or 40 hours, and others not till as many days after the attack, especially when they were removed on board from the noxious air of the island. A great proportion changed in a few days to a bright yellow; some to a leaden colour. Other cases terminated fatally, in a very rapid manner too, without the slightest alteration in that respect. Generally, however, the change of colour indicated great danger. Vomiting of black bilious stuff, resembling the grounds of coffee, frequently commenced early, and continued a most distressing symptom. * * * * * Hæmorrhage from the mouth and nose seldom occurred; in two cases, which terminated fatally, the blood did not coagulate, but tinged the linen yellow. * * * Two kinds of eruption appeared about the lips; one such as we often see at a decline of common fevers; the other consisted of small black or brown spots round the lips, and was likewise a dangerous, indeed a fatal, symptom. With this eruption, the teeth, tongue, and fauces generally became covered with a brown or black coat, and the breath intolerably fætid. * * * In those cases which occurred on board, and where the patient had not slept on shore at Edam, the symptoms were much milder, and the fever resembled more the bilious remittent of other parts of the East."

"Never," continues Mr. Shields, "was there a disease so deceitful as this fever. I have frequently seen instances where every symptom was so favourable, that I could have almost pronounced my patient out of danger;* when, all at once, he would be seized with restlessness, black vomit, delirium, and convulsions, which, in a few hours, would hurry him out of existence! * * * No constitution was exempted from the assault of this fever. It seized with equal, or nearly equal, violence on those who had been many years in India and on the most robust and plethoric, or newly-arrived European. Even the Dutch officers and Malays, who had been drawn from different parts of Java, and whom we had prisoners at Edam, fell victims as fast, or nearly so, as the English."

The mortality was frightful. Almost every individual, who slept on shore at Edam, perished. The disease was certainly not infectious or transmissible from one person to another; "for," says Mr. S., "on our raising the blockade of Batavia, great numbers of sick, in every stage of the fever, were brought on board from the hospital at Edam, yet not a single

---

* Mr. Shields mentions one case, where the patient felt himself on the 2nd or 3rd day so much relieved that he called for some mutton broth and sago, which he eat with a good appetite; he spoke naturally, and was in good spirits. Towards evening, a change took place, and he was a corpse before morning! Such an occurrence is frequent in the yellow fever of the West Indies. A feeling of hunger in the delusive lull, that so often occurs about the 3rd day or so, is well known to be a frequent forerunner of a mortal change.

nurse or medical attendant of any description ever suffered the slightest attack; nor did any circumstance transpire that could in the least favour the idea of contagion (infection), notwithstanding that the great accumulation of sick on both decks rendered it a matter of impossibility to separate them completely from those who were well, nor at all times to prevent a considerable generation of effluvia."

So much for the phenomena exhibited during the life of the patient in a malignant form of the remittent fever of an intertropical country. Let us now compare the *post-mortem* appearances, as detailed by Dr. Copland, in the two diseases between which there is so close a resemblance.

*Yellow Fever.*—"In the most malignant and rapidly fatal cases, the *muscles* are softer and flabbier than natural, of a dirty or dusky hue, and are easily broken down by pressure. The substance of the *heart* is similarly changed. * * * The *liver* is changed chiefly as regards its cohesion and degree of congestion. It is almost always softer and more friable than natural; in some cases congested, in others pale. * * The *spleen* and even the *pancreas* are somewhat softened; and the former frequently congested. * * * The epithelium of the *digestive mucous* surface seems to be more or less detached in the several portions of the canal; and the mucous membrane is softened and readily separated from the adjoining tissue. The *follicular glands* are not prominently affected, further than being somewhat enlarged in some instances.

"In those cases which present congestion of the chief organs, as of the brain, lungs, auricles of the heart, liver and kidneys, slight *serous effusion*, sometimes sero-sanguineous, is occasionally also found in the chief cavities, particularly the *pericardium* and *arachnoid*, and but rarely in the *peritoneal* and *pleural* cavities." Vol. I., p. 143.

*Malignant Remittent Fever.*—"The substance of the *heart* is frequently soft, flaccid, and readily torn, the cavities being occasionally dilated, more especially after the adynamic states of the disease. * * * * The *liver* is usually injected, remarkably softened, of a dark colour, friable, and sometimes enlarged. The *spleen* is often so soft as hardly to admit of being handled."

The *digestive mucous surface* is softened, injected, ecchymosed, of a dark hue, and sometimes thickened, abraded, or even ulcerated in the lower parts of the canal. The *mesenteric* glands occasionally, and the *pancreas* more rarely, are enlarged or otherwise changed.

The *lungs* are sometimes congested, infiltrated, condensed, or inflamed. The *pleura* and *pericardium* often contain some dark sanguineous serum. * * * * The changes *within the cranium* consist chiefly of congestion of the veins of the pia mater and sinuses, with a fluid dark blood, and sometimes of *effusion of serum* into the ventricles and between the membranes. Vol. III., p. 950.

It may not be unprofitable to introduce here, for the purpose of comparison with the details now given, the most remarkable *post-mortem* appearances described by our author as being usually found in the bodies of those who die from a rapidly fatal attack of Ardent or Seasoning Fever.

"The *digestive mucous surface* is studded with numerous dark or ecchymosed spots, from which a fluid black blood seems to ooze. The *liver* is frequently congested, sometimes larger and softer than natural, and of a dark colour, owing to the quantity of black blood in its vessels. The *spleen* is somewhat enlarged, soft, and friable; and the omentum injected. The serous as well as the mucous surfaces, especially in the abdominal cavity, often present livid or dark patches. The *blood* is everywhere fluid, black, and dissolved. The internal surface of the *heart* and large vessels, both arteries and veins, was of a dark red or livid tint in a few cases which I examined; but this point requires further investigation." Vol. I., p. 978.

The most inattentive reader cannot fail to observe how remarkably alike the post-mortem appearances are in the three diseases to which reference has been made. It is surely unnecessary to add a word in the way of comment. We shall therefore now proceed to make a few remarks on a point connected with the history of Yellow fever, on which great stress has been laid, by many of the ultra-infectionist party, as indicating, they contend, a marked difference between it and all malignant remittents.

That genuine yellow fever ever exhibits any thing like distinct remissions in its course is emphatically denied by Dr. Copland; and so convinced is he of the truth of his opinion, that he even goes so far as to assert that, whenever this feature is observed, the existing disease may at once be set down as not the genuine pestilence. This mode of reasoning has certainly the merit of boldness; for it unmistakeably declares in very simple language the point to be determined. So far it is well; there can be no mistake as as to the *q. e. d.* It is obvious, however, at the same time, that the difficulty of disproving the position is considerable; for the argument resolves itself into a mere unproved assertion. It would be easy, indeed, to adduce a multitude of most respectable authorities in flat contradiction of the statement in question; but these we shall straightway be told, are of men like Bancroft, Jackson and other non-infectionist writers, whose object it is to shew that there is no essential or infallible distinction between malignant remittent, and genuine yellow fever. Dr. C. forgets, however, to tell his readers that some of the most zealous advocates of the infectiousness and importability of the disease have declared the very same thing. He knows, for example, that Dr. Arejula, who saw so much of the yellow fever in the south of Spain in the beginning of the present century, and whom he is so often glad to cite as a most competent witness respecting it, expressly says that, the disease "without doubt deserves the name of remittent fever." Dr. Balmis too, whose experience was very large, called the disease, as he saw it at Cadiz in 1800 (the genuineness of that epidemic is more than once admitted by Dr. Copland), "a putrid malignant remittent." The same view was held and expressed by various others of the Spanish physicians, almost all of whom, it is to be remembered, were out-and-out infectionists and importationists. Several of them, Arejula among the number, have even asserted that, the disease sometimes assumes an intermittent form. "In conclusion," we quote from Dr. Gillkrest's excellent article in the Cyclopædia of Medicine, where ample particulars respecting the opinions of the Spanish physicians will be found, "on this part of the subject, it may be stated that the records of the Gibraltar yellow fever epidemics furnish the following names in support of the fact, that remissions not unfrequently take place in this disease—Drs. M'Mullin and Browne, Messrs. Sproule, Wild, Martindale, Amiel, Daw, Donnett, Humphries, Lee and Hugh Frazer." Dr. Copland himself admits in one passage that, "slight exacerbations are sometimes remarked in the evening and ameliorations in the morning; but these are rarely so considerable as to amount to remission." True; but then does he not over and over again tell us that, in the bad forms of endemic fever, the remissions are sometimes scarcely, if at all, perceptible? Nay; according to his own statement, this is of not uncommon occurrence; for, in one part of his narrative, we read that "the passage of that type (malignant remittent) into the continued is extremely frequent, especially when vital organs become more and more implicated, and when the disease increases in prevalence, so as to assume an epidemic character; the fever, with this change of type, presenting in the worst cases the chief features of the Hæmagastric pestilence." Again; "there is generally much difficulty in distinguishing the malignant remittent of Africa from this pestilence, owing to *the imperfect remissions of the former*, and to the presence, at an advanced stage of fatal cases, of many of the symptoms characterising the early and rapid progress of the latter." It would be easy to quote passage upon passage, from the writings of our most experienced army and navy medical officers in the West Indies, in illustration of the point in question. One will suffice: it is an extract from the official report of Sir D. Dickson, the late able physician to the Leeward Island Fleet, and is quoted in Dr. Bancroft's work.

"At Barbadoes and Antigua, I had generally seen the disease of an ardent *continued* form, and did not fully understand why authors talked of a bilious *remittent* yellow fever, until after the capture of the French and Danish islands. But the anomalies of fever, the shades and changes which it assumes according to the intensity of the exciting causes (while these were purely and solely local), the state of predisposition or the spot of residence, could no where be more strongly portrayed than in the destructive epidemic of Marigalante in the Autumn of 1808, from the most concentrated marsh miasmata; when the different types of fever were controverted into each other, of the worst and most aggravated species I have ever witnessed. Some were affected with the highly concentrated yellow fever in the *continued* form; others with comatose *remittents* or *intermittents*, the exacerbations of which were so violent as to carry off a patient in two or three paroxysms; while others sank into a low protracted character of fever resembling *typhus*."

Need more be said to shew how utterly vain it must be to attempt to build any satisfactory distinction between malignant Remittent and genuine Yellow fever upon the character of the continuedness or periodicity of the febrile symptoms? Surely not. We shall pass on to the investigation of another point, on which also there has been no inconsiderable discordancy of opinion among medical writers.

The point to which we allude is that respecting the non-liability to second attacks of yellow fever, and the value of this character or attribute as a diagnostic sign between it and the other fevers with which it is apt to be mistaken. Dr. Copland goes so far as to assert that one attack of the genuine disease "protects the system more decidedly from a future seizure than even Small-pox or Scarlet Fever from a second attack of these maladies." This is certainly a somewhat exaggerated statement; but we shall concede its truth just now for the sake of argument; and we admit, at the same time, that second attacks and relapses of common marsh Remittent fever are very frequent. Was the question simply and entirely between this fever on the one hand and well-marked severe Yellow fever on the other, there could be no difference of opinion upon the question we are at present considering. A second attack of the latter is a very rare occurrence indeed, and that of the former is a very common one. But then have we not already been told that second attacks of the Ardent or climate fever are also very rare; and that, when such an occurrence takes place from the circumstance of the person having intermediately returned to a temperate climate, this disease generally assumes on its second invasion a remittent type? This admission has very important bearings upon more than one of the controverted points in the present discussion. For example, it is very confidently stated by our author, when speaking of some of the epidemics of yellow fever at Gibraltar, that none of the garrison enjoyed immunity from its attacks, except those who had had the disease before either in the West Indies or in Europe. Now we should like to know how he learned so satisfactorily that all these protected persons had had the genuine disease, and not that very many of them, probably a large majority, had had, on the previous occasion referred to, the mere seasoning fever of new comers into a tropical climate? Not a particle of evidence is adduced to prove the fact as stated; its truth rests solely upon the testimony of the parties themselves. Can any impartial person, acquainted with the history of disease, be satisfied with such evidence? We have already seen how difficult it is, even for a medical man, to discriminate the one distemper from the other. What reliance then can be placed upon the mere affirmation of unprofessional persons as to the real nature of a febrile disease, which they may have experienced at some previous period? Is it not the case too, according to our author's own shewing, that the majority of practitioners in the West Indies and elsewhere are continually mistaking other fevers for the genuine pestilence? If the patients, therefore, derived their information from their medical attendants, the probability therefore is that it was not the true yellow fever that they had suffered from.

And this probability is surely much increased, when we call to mind Dr. Copland's opinion that, while the Ardent, the Bilio-gastric, and the various forms of Remittent fever are endemic, and therefore of continual occurrence, the Yellow fever occurs only occasionally (at intervals often of several years), and then generally as an epidemic.*

Is there not therefore the strongest presumption that, of the 6000 persons who, during one of the Gibraltar epidemics, escaped the disease in consequence of having had it previously in the West Indies or in the South of Europe, the previous disease was in short nothing more than the climate fever of these countries? At all events, we must have something more than the mere assertion of the fact, before we can admit our author's opinion, that in all these 6000 cases the primary fever was his genuine pestilence, as contradistinguished from all other intertropical fevers.

Here too it may be worthy of notice that, although relapses and second attacks of common Remittent fever are abundantly common, we find no evidence to shew that second attacks of the malignant form of this fever have ever been met with. It may suit the convenience of a one-sided dis-

---

* "I doubt much," says Dr. C. "his (Dr. Bancroft) having seen a case of true hæmagastric fever, when he published his Gulstonian Lectures, delivered at the College of Physicians in 1805." Are we to regard this remark as an illustration of the comparative rarity of the disease; or is it merely a little explosion of angry feeling? On either view, it will not surely aid our author's argument.

putant to make the general assertion respecting remittent fevers in the lump; but this will not do with any one accustomed to sift the ambiguities of medical testimony. Not only have we no proof of any second attacks of the aggravated form of remittent fever, we mean that in which there is yellowness of the skin, and any tendency to black vomit; but there is also an utter lack of evidence to prove that any one, who has recovered from the disease in question, has ever subsequently been attacked with yellow fever.

It is altogether amusing to watch the conjectural shifts, to which the adoption of extreme and exclusive opinions upon an intricate question of medical evidence leads even the most experienced. For example, it is laid down by Dr. Copland, with all the confidence of a well-ascertained fact, that, while the Ardent fever is liable to be followed, upon recovery, by different forms of periodic fever, the genuine Yellow fever never is; but then we find this very important admission at the same time, that "as it (the latter) usually prevails in situations where remittent fever is endemic, and in seasons when this is most prevalent and malignant, recovery from the malady is extremely likely to be followed by an attack of the latter; and, more than this, the one disease is very liable to be mistaken for the other." This is certainly a very easy way of getting rid of an objection, that has often been urged with force against the opinion of the essential and specific difference between Yellow and Remittent fevers; but will it satisfy any inquisitive mind? Again, we read in another passage that "most of the instances of recovery which we hear of from the black vomit are recoveries from those states of malignant bilious or remittent fever which have been confounded with the pestilence." Here then we have another element of perplexity introduced into a subject, intricate and involved enough in all conscience; for is it not possible that the few of the 6000 exempt persons, mentioned above, who may have been previously affected with a fever attended with black vomit, had not had the genuine yellow fever, but merely a malignant form of the endemic remittent? It is obvious, therefore, that according to the testimony of Dr. Copland himself, we cannot be warranted in admitting, as an argument for the non-liability to second attacks, the very strongest of all the cases that he is able to adduce. As we shall afterwards have occasion briefly to allude to this matter again, in commenting upon some of the statements of Sir William Pym, we need not say more upon it at present.

Before proceeding to make any remarks on the alleged essential and invariable *infectiousness* of Yellow Fever, we shall avail ourselves of this opportunity to notice Dr. Copland's views respecting its ætiology. The true and only cause of the disease is declared by him to be "an infectious or contagious poison which, however formed originally, infects the healthy by contaminating the air immediately surrounding those already affected, or which, being absorbed and retained by other bodies, is afterwards given out from them on exposure to the air, thereby contaminating and infecting the air and adjoining objects." This morbific poison is alleged to arise "independently of endemic or terrestrial sources or malaria," and, in almost all cases, to be an emanation or effluvium from the body of a person already affected with the disease. Dr. C., as a matter of course, feels at a loss how to account for the origin of the first case during an epidemic visitation; for it is obvious that there must be a *fons et origo*

*mali* somewhere, independent of personal transmission. But even this consideration will scarcely shake the settled conviction of his mind as already expressed; nay, it even serves, in a most unexpected manner, to establish it the more; for thus he reasons:

"It has appeared in one or two isolated persons who had not previously breathed the foul air of sick wards or apartments; nor visited the places in which either the sick or the healthy had been confined; nor inhaled the effluvium from decomposing exuviæ and animal substances; and thus, by the exclusive process of reasoning, we have left only that cause to which I have imputed it; and by means of which it is as undoubtedly propagated and perpetuated as any other malady whose infectious properties are admitted." P. 177.

But we must not forget to state that, while our author rejects the idea of the true pestilence ever originating from the operation of any local or endemic malaria, he is not without his own hypothesis on this difficult subject. It is broached in the following passage:—

"That the contamination of the air, especially when it is humid, warm, and close, either by other fevers, or by other maladies, or by a number of persons previously in health confined in it, will take place, so as to produce fevers of a malignant character, more especially that fever which I have called Putro-Adynamic (see Fever, §§ 484—496), I have shown when treating of that malady (§ 496); but satisfactory proofs are wanting of this pestilence ever having originated in this way. Since my visit, however, to several places in Africa, and knowing the very limited space in which a large number of slaves are often confined, both on shore and in slave-vessels, I entertained the idea that this pestilence or its seminium, or specific infection, had been generated originally by the congregation of negroes in a close atmosphere, or is generated *de novo* by this race when placed in the circumstances now stated; and that, although it affects them in a comparatively slight manner, it is most particularly baneful to the natives of cold countries; as small-pox is comparatively mild in the white races, whilst it is most pestilential and fatal amongst the negroes. This opinion, entertained since 1817, I have endeavoured to ascertain the truth of whenever I have had an opportunity of making any inquiry respecting it; but the evidence is not sufficient to establish this as the source of the infection. The following, however, may favour the truth of this idea. A small vessel in which I was a passenger was anchored, in May of 1817, a short distance from Sierra Leone; and the ship's boat with four of the crew was bringing me on board when a tornado suddenly overtaking us we took shelter on board of a ship recently brought into the harbour full of slaves, and near which we were at the time. The men belonging to the boat took shelter down between decks. I remained under a small poop on the quarter-deck. All these men in two or three days were seized with this distemper, the vessel having just put to sea, and I escaped. The sick men were constantly kept on deck, free ventilation was enforced, and every possible precaution under the circumstances was used, and no more were attacked." Vol. III., p. 175.

Here the distinct impression is left upon the reader's mind that it is only the black population that is capable, under the circumstances supposed, of originating the disease. But that malignant and pestilential fevers of an highly infectious nature, and exhibiting all the characters, if not possessing the nature, of the true yellow fever, may be generated in hot and sickly climates by the crowding together of other races of men without due ventilation, had already been admitted by Dr. C.

"Some of the epidemics of yellow fever said to have occurred in the West

India Islands, and in parts of the American continent, have been much milder than the visitations of this pestilence have been in Spain, during the early part of the present century. But it is by no means fully ascertained whether all the epidemics observed in the western hemisphere were actually the true yellow or hæmagastric fever, or merely an unusual prevalence of endemic or remittent fever, rendered more continued by intensity of attack, predisposition of the affected, and other circumstances. No doubt several of these epidemics were the pestilence under consideration. Their symptoms, remarkable prevalence, and fatality proved that some of them were this distemper; but others were of a different nature; and probably some of them resulted from the crowding of a number of human beings in a confined space, either in barracks, or in transports, or between the lower decks of ships of war, in a high range of temperature, and without sufficient renewal of the air." Vol. III., p. 149.

How much it would have tended to elucidate this perplexed subject, if Dr. Copland had specified which of the epidemic visitations, alluded to, he regarded as belonging to the genuine pestilence, and which he looked upon as belonging to other forms of fever! As the matter stands, it is not possible to establish any comparison between the one set and the other, and thus seek to discover their prominent points of difference, if any such really existed in nature. But, without dwelling upon this point, and adverting only to the (supposed) negro origin of the genuine disease, one or two difficulties will immediately suggest themselves to the reader's mind. How comes it that we do not hear of the disease (so, at least, it is asserted by our author) " in the eastern hemisphere, and on the shores of the Pacific?" Have not many crowded slavers arrived, at different times, at the Mauritius and the Isle of Bourbon? and is there not a regular slave trade carried on in the present day at the island of Madagascar, where, nevertheless, the true yellow fever is said never to have been known? Apart, however, from these considerations, is there, we would ask, any other example of an essentially specific disease, a disease *sui generis*, and proceeding, as it is declared, from a morbific *seminium* or virus analogous in its nature and peculiarities to that of Small-pox or of Measles (for all this is asserted), being produced by, and originating from, the mere accumulation of human beings under any circumstances? Are any of the Exanthemata ever so developed? or is there any reason to believe that the other two pestilences described by Dr. Copland—viz. Epidemic Cholera and the Plague—ever originate from the cause mentioned, apart from the operation of certain endemic malaria? Again, is it not somewhat surprising that a disease, (supposed to be) generated by negroes, should exert its infectious properties comparatively so exclusively upon the white races of mankind, and more especially upon those who have recently arrived in a tropical region? Is there any analogous instance of such a limitation, on the part of an essentially infectious disease? We surely know of none. Small-pox commits its ravages with equal fatality among all races of the human family, who have not been protected by vaccination; and the same thing may be said of Measles and Scarlet fever. But it is very different with the Yellow fever. The negro races, it is well known, although certainly far from being exempt from its invasion, seldom suffer so severely from it, as those whose systems are less acclimated to the heat and malaria of a pestiferous climate. Does not this circumstance alone point to some connection, in nature as well as in cause, between this and the ardent or seasoning fever? We have already seen how closely a malignant case of the latter resembles, in all its features, the most fatal yellow fever.

But passing from these considerations, we should like to be told how the Malignant Remittent, when it assumes a continued type and acquires infectious properties, is to be distinguished from the genuine Pestilential disease. This dangerous modification of the former is apt to take place in certain seasons, when the endemic fever prevails in an epidemic form, and especially when the vital organs and the circulating fluids are more seriously affected than usual. Under such circumstances, it is unquestionably liable, as Dr. Copland allows, to become infectious whenever a number of the sick are crowded together in badly-ventilated places, "during very warm and humid states of the air, so as to contaminate the surrounding atmosphere, and thereby either to superadd the additional cause of a morbid effluvium, exhaled from the sick, to existing marsh miasma, or to generate a morbid poison or vapour which is of itself capable, independently of marshy or other miasmata, of infecting the healthy and disseminating the distemper." "That a superadded quality," our author goes on to say, "or at least a change of character, should result from the circumstances just alluded to, may be rationally inferred; for the aggravation of symptoms and the development of new features in these altered circumstances have frequently been observed, are undoubted, and are the chief sources of much of the differences of opinion and of the discussions which have appeared, since the end of the last century, on the subject of the Yellow fever." Yet he afterwards, in more passages than one, appears to argue as if the possibility of the endemic fever ever becoming infectious was entirely out of the question. For example, when combating the doctrines of the venerable Jackson on the subject, he writes thus :—

"He (Dr. J.) remarks that 'there is not one practitioner in one hundred who has resided for years in the West Indies, who believes that the concentrated endemic of that country, usually called the yellow fever, is a disease which possesses the power of propagating itself from person to person within the tropics.' Certainly there is not. It is well known that all the writers on West India diseases during the last and present centuries admit this, but many of them—nay, the majority,—also admit, what is the fact, that the severe endemic of that climate is not this pestilence; that the former is liable to be mistaken for the latter; and that both are often confounded together, although they are as distinct, indeed more distinct from each other, than measles and small-pox. And in this Dr. Jackson errs with the minority, using at the same time terms which involve a theory, or mean nothing. Thus his 'concentrated endemic' must either mean the more malignant form of remittent, which I have described from frequent observation of it in warm climates, in the *art.* Fever, by the name of *malignant remittent,* and which I know well *is neither infectious,* nor the pestilence now under consideration, the differences between which have been long since pointed out by many very intelligent and experienced observers." Vol. III., p. 164.

Here Dr. C. makes the broad unqualified assertion that the Malignant Remittent (which, we have already seen, he admits not unfrequently assumes a continued type) does not exhibit infectious properties; and, in another passage a little further on, we find him laying hold of an extract from Dr. Jackson's writings, wherein allusion is made to the effects of crowding the sick together upon the diffusion and aggravation of the disease, to found a charge either of ignorance or of unfairness against this most esteemed physician. We leave the reader to judge for himself from its perusal:

"The yellow fever," says Dr. Jackson, "during the reign of epidemic influence often strikes like a pestilence by the mere concourse of people in a close place; and if a mass of sick persons be collected into an hospital during the epidemic season, the common emanations from the sick bodies, whether saturated with contagious particles or not, often act offensively on those who enter the circle, and often appear to be the cause of the explosion of a disease which, without such accessory or changed condition of the medium in which men live, would have probably remained dormant for a time, and perhaps for ever. The instances of persons who have lived in apparent good health in simple epidemic atmospheres, and who have become sick soon after they entered into the circle of a crowded assembly, or the crowded wards of an hospital of sick, are numerous, and so well marked, that they stagger, on a superficial view, the opinion here contended for, of the non-contagious nature of the yellow fever.' To be sure they do, and being admitted by Dr. Jackson they become evidences of infection as strong as 'proofs from holy writ.'" P. 165.

Indeed! Has not Dr. Copland himself told us that the Malignant Remittent may become infectious, "when a number of the sick are crowded together in badly-ventilated places, during warm and humid states of the air?" and has he not admitted the very same thing in respect of his "bilio-gastric fever," another denizen of those very regions where the yellow fever is most apt to be met with?

We therefore see, from the data supplied by our author himself, that the character or attribute of *infectiousness* cannot be fairly set down as a diagnostic or discriminating feature of the genuine Pestilential fever; and thus the presumption that this attribute is not a uniform or necessary, but rather a contingent and occasional, property of the disease appears to be rendered more and more probable. We shall afterwards see how far this view is borne out by the history of the Eclair fever. Meanwhile, let us keep our minds as free as possible from all extreme and exclusive opinions, taking part neither with those who can see no cause save that of personal transmission in the work of morbific dissemination, nor yet with the ultraists on the opposite side, who have unguardedly asserted that Yellow fever is never propagated in this way.

What has been said by an able writer in the Dictionnaire de Medecine et de Chirurgie, *art. Contagion,* of the labours of the late indefatigable M. Chervin in reference to one epidemic of the pestilence, may be applied with strict truth to almost every other one of which we have any published account:

"Observe, in regard to this last subject, (viz. the error of attributing to infection what is often referrible to local causes), what occurred respecting the yellow fever epidemic of 1821 at Barcelona. Read the work of the French medical commission appointed to examine into that epidemic, and it will be impossible for you (admitting as true the statements therein contained), not to admit the existence of infection. But afterwards, when you have read the documents collected by Dr. Chervin with a degree of zeal and patience truly admirable, you will be convinced that the circumstances, which led you to be of the same opinion with the commissioners as to the reality of infection, are anything but conclusive."

So necessary is it to weigh and balance all things together, before we form our opinion.

There is a subject, connected with the occasional outbreaks upon a large scale of the Yellow fever, that deserves much attention, but which, like every other point in the history of this perplexing disease, is surrounded with difficulties. Is it truly endemic, or does it occur only occasionally, in those countries where it usually appears? in other words, does it exist there in a partial or sporadic form in all years, just as the malignant Cholera does in the East Indies, or as the plague does in Egypt? or is it entirely extinct, save and except when it appears epidemically, as is usually the case with exanthematous fevers, viz. Small-pox, Measles or Scarlatina? Dr. Copland seems to adopt the latter opinion; for he says, when pointing out the diagnostic characters of the inter-tropical fevers that are so apt to be mistaken for each other, that "the *remittent* is endemic in warm climates, and in several temperate countries in warm seasons, especially those abounding with the sources of malaria: the *ardent* fever occurs only among persons who have recently arrived from cold or temperate climates into a very hot country; and *true yellow* fever appears only occasionally; and then the infection may either extend to a few only, the circumstances favouring its diffusion not existing, or to great numbers, the disease thereby becoming epidemic. Thus the *first* and *second* of these fevers are always occurring, especially the first; the *third* seldom, or after long intervals."

Now here is a declaration in express contradiction of the opinion held by at least nineteen-twentieths of the medical men practising in the West Indies. That not a year passes over without a few sporadic cases, at least, of yellow fever occurring in those places that have usually been the scene of its epidemic visitations, is admitted by an overwhelming majority of those who have resided long in such districts. Indeed, we strongly suspect that Dr. Copland himself, notwithstanding the above quoted opinion—which, we should mention, occurs in the 1st volume of his Dictionary, published 12 years ago—is now by no means satisfied of its correctness; for in the very last Part, when alluding to an outbreak of the disease at Port Royal Jamaica, we find him saying;—"it should be premised that the infection of this pestilence had lurked for several years, *or even longer*, in the most frequented sea-ports of the West Indies, as Port Royal, the Havannah, Vera Cruz, &c." We may mention also that some of the most zealous infectionists, as well as others of the opposite party, have strenuously maintained that the disease is never entirely extinct in these and such-like places.

The casual mention of this point naturally leads us on to the consideration of another, which has given rise to a vast deal of learned and elaborate discussion. The question to be determined is, whether the so-called genuine Yellow fever has been always an indigenous product of the West Indies, or whether it was conveyed thither, towards the close of the last century, from Bulam on the western coast of Africa; the malignant fevers that may have previously existed in the western hemisphere being, on this supposition, believed to have been only aggravated forms of the endemic remittent, but not belonging to the true specific pestilence which the appellation of the Bulam or black-vomit fever should, it is said, be employed to designate. Few indeed, in the present day, entertain the latter of these opinions; but, when it was first brought forward in 1794 by Dr. Chisholm, the confidence of his assertions, and the fortuitous outbreak of the pestilence in many of the West India islands after a lull of several years, had the effect of making a good many converts to his new and startling doctrine. Sir William Pym has always been one of its warmest espousers. We are glad, however, to find that Dr. Copland, albeit so energetic an advocate of the specific and essential difference between the genuine yellow fever and every form of endemic tropical fever, repeatedly and unequivocally acknowledges that the former had frequently broken out not only in the West Indies, but also in different parts of the United States, during the last, and even during the 17th, century; and, although he refers more than once to "the re-appearance (in 1793) of the malady in the West Indies after an immunity from it during many years," he judiciously avoids all allusion to the often-repeated assertion by certain ultraists that it was then imported for the first time from Bulam, on the African coast, by a vessel called the Hankey. As he therefore, with all his strong bias on the subject of importation, evidently considers the arguments of these gentlemen utterly unsatisfactory, by his not even so much as alluding to the name of this vessel, it may be very fairly presumed that the point in question is altogether untenable, and is not likely ever again to become a topic for much difference of opinion.

That there was an outbreak of the disease, in an unusually severe form, in the West India islands in 1793, after many years of comparative immunity or rather mildness, cannot for a moment be doubted. The question of dispute is, whence did it come? Dr. Copland, as we have already stated, wisely avoids giving any definitive or peremptory reply. He merely says that it *re-appeared.* It broke out, at that time, first in Grenada; but not a word is said by him as to the producing, far less of any imported, cause of it in that island. He leaves indeed the impression on the reader's mind, although he does not make any positive assertion, that it spread thence by infection to the other islands of the West India Archipelago, and to different parts in the United States. Without canvassing this opinion at present, we shall merely remark that the course, which the disease seems to have pursued in its travels, appears to be anything but very consistent—seeing that there was no restriction to free intercourse between the different islands—with the supposition that personal infection or direct transmission from one individual to another was the sole or principal agent in its dissemination. The pestilence broke out at Grenada in February, 1793. In July of the same year it made its appearance in Dominica, and in the very same month, at Philadelphia,* many hundred miles distant. Barbadoes remained unaffected until the beginning of 1794, and St. Domingo did not suffer until late in the course of that year; when it seems to have reached most of the West India islands. But it would be unprofitable to attempt to pursue the examination of a subject, on which such contradic-

---

* There seems to be no little difference of opinion as to when the disease made its appearance at New York, during this epidemic. Dr. Copland quotes the testimony of two gentlemen, Drs. Charlton and Bard. The former says that it appeared there in 1793, while the latter states he never saw a case of it until 1795.

tory evidence has been brought before the public. Even with respect to the cause of the occasional appearance of the pestilence at Sierra Leone and other parts of the west coast of Africa, no little contrariety of opinion has been expressed by different medical writers. While Chisholm and Pym maintain that Bulam is the true birth-place and nursery of the disease, the late Mr. Fergusson wrote an article* to prove that it is unquestionably indigenous in Sierra Leone, 130 miles distant, and that it is generally transported from that pestiferous place to other points on the African coast. On the other hand, of the two epidemics which prevailed there in 1823 and 1829, Dr. Copland expresses his opinion that they (at least the latter) did not originate in the colony, but had been imported:—from what place, he does not say. He rests his opinion on the report of the then governor, Major Ricketts, "who," our author says, "has thrown light upon a subject which they (the colonial surgeons) have confused and mystified." Allusion must here be made to Mr. Boyle, who wrote a work upon the subject.† In that work, after adducing a variety of details, this gentleman says:—"The foregoing information appears to be so conclusive as to the origin and original nature of the disease, that further discussion upon these points must be unnecessary. That there are differences in the accounts given by the natives is true; but they do not amount to discrepancies of such a magnitude as at all to invalidate or shake the one leading supposition and conviction, *viz.* that the epidemic fever, which raged so fatally in Freetown in 1829, was immediately caused by peculiarities in the seasons, originated in the interior, was borne to Freetown by the North-east winds, and, in its primary and true character, was not contagious."

With respect to the former epidemic, we learn from Dr. Gillkrest that Mr. Showers (who was resident Colonial Surgeon from 1816 to 1826) has stated that, when the fever broke out in 1823, it was supposed by some to have been imported thither from the Mediterranean, by a vessel called the Caroline! Mr. Showers, however, was of opinion that the disease proceeded from the atmosphere. Well may Dr. Gillkrest say, when alluding to this matter, that "to those, who had been led to believe that the true black-vomit fever had been not unfrequently *exported* from the coast of West Africa, its reputed birth-place, this visitation as a perfect stranger, and its alleged importation from Europe, must appear somewhat strange."

It is remarkable how unwilling the residents of a place often are to allow that a pestilential disease has originated among themselves: they will generally assert that it has been brought *ab extra* to them.

Dr. Copland's views, as to the origin of these two epidemics at Sierra Leone, may be gathered from the following passage:—

"Although persons belonging to the colony had visited places adjoining where the pestilence was raging at the time, had returned to the colony, and were immediately afterwards attacked and died, other cases of the disease following upon these admitted to have been the first; and although ships, more especially slave-ships with sickly cargoes of human beings, arrived at the colony just before and at the time of the outbreak of these epidemics, some of the writers on the subject contended that the distemper had arisen from malaria brought from a distance

---

* *Medical Gazette* for August 31, 1839.

† *Account of the West Coast of Africa, &c.* London, 1831.

by the winds, and others concluded that it had travelled from the interior of Africa to the coast—a sufficient admission of infection; whilst the more observant of the residents believed in its introduction through one or other of the channels just indicated, or through both." Vol. III., p. 172.

The reader will naturally enquire whence came the pestilence to the *adjoining places*, from which the colony is supposed to have received it? The mention of " the slave-ships with sickly cargoes of human beings," has reference doubtless to the hypothesis of the origin of the disease, which has been noticed in a preceding page. From the coast of Africa, we now pass to the shores of Europe.

Without entering upon the angrily-disputed field of enquiry as to the probable origin of the several epidemics of yellow fever, which have made their appearance in different parts of Spain during the course of the present century—and what good could result from such an investigation, when we have not a single datum or position that has not been flatly contradicted?—one or two remarks may not be without some interest and even profit to the reader.

The evidence, adduced by Dr. Gillkrest to shew that many parts of the Peninsula had repeatedly suffered from visitations of this fever in the course of the last and the preceding centuries, and probably also at a still earlier date, cannot, we should think, be resisted by any unprejudiced person. Sir Gilbert Blane himself expressed his belief that it was known at Cadiz in 1764; and Dr. Copland not only alludes to this case, but also mentions that the pestilence " appeared at Lisbon in 1723, black vomitings being the most prevailing and fatal symptom." With such an admission, it seems rather strange that he should afterwards talk of " the first appearance of the pestilence" at Gibraltar in 1804; and, notwithstanding this too, the particulars mentioned by Dr. Gillkrest of the visitation in 1798 (not to allude to any earlier ones), when " Dr. Harness, then physician to Lord St. Vincent's fleet, and afterwards one of the Commissioners to the Sick and Hurt Board, declared the fever to be precisely the same he had seen in the West Indies;"—a statement that is confirmed by Dr. Monro in his Work on Diseases of Armies.* It may be mentioned also that Dr. Copland himself acknowledges that the pestilence was at Cadiz and Xeres in 1800, and at Malaga in 1803, places not above forty or fifty miles east and west from Gibraltar. He says that the escape of the " Rock" in these years was owing to the quarantine regulations that were adopted. Yet he is obliged to confess that, in spite of these regulations enforced too, be it remembered, under the vigilant eye of Sir W. Pym then superintendent of quarantine at Gibraltar, it found its entrance in the following year, 1804.† Such was the case also in 1810 and 1813; and again at the last visitation, that of 1828; " the quarantine," says our author, in reference to the vessel called the Dygden, that was believed to have imported the disease from the Havannah, " appears not to have been rigidly enforced." Alas!

---

* We might allude also to the well-authenticated case of the yellow fever prevailing on board of the Bedford man-of-war, 74, in the bay of Gibraltar in 1794.

† Dr. Copland tells us that Sir W. Pym met with *one case* of the distemper in this fortress (Gibraltar) in 1803.

the watchmen seem always to be asleep, at the very moment when the enemy is ready to enter!* Dr. Copland gives a minute and most circumstantial account of the exact mode in which the pestilence was introduced by that unfortunate vessel, and he even traces each successive step in its stealthy progress "from the smuggler and washerwoman to whom the foul clothes had been sent," the first affected, until its full-blown developement, when it prevailed to a fearful extent in almost every part of the garrison. It is surely very strange, to use the gentlest expression, that he does not even so much as allude to what Dr. Gillkrest has written on the subject, although this gentleman was on the spot at the time of its outbreak, and had an opportunity of watching its rise, progress and termination. Courtesy alone might have prompted Dr. C. to have referred to the statements of an honourable opponent, writing in what may be fairly deemed a rival work. Any one, who reads Dr. Gillkrest's apparently most fair narrative of this epidemic, will hesitate before he receives our author's circumstantial details, if he does not at once wholly discredit them. If we are to give credit to Dr. Gillkrest's statement that "the failure of proof as to the importation of the disease has been admitted by the Army Medical Board in England, to whom a copy of all the proceedings of the Gibraltar Commission was sent for examination," can we require any further reason to make us pause in our decision upon the matter, and not hastily to adopt an opinion, one way or the other, merely because it may happen to coincide with our previous and favourite notions? These remarks must suffice upon this part of our subject. The fair and legitimate conclusion may surely be asserted to be that the Yellow fever was not a *nova pestis* introduced, for the first time in 1793, into the western hemisphere from the African coast, and re-exported from the former, in some subsequent year, into the south of Europe. Yet this opinion is still most tenaciously held by one, if not by more, of the veteran writers upon the disease! We are glad to find, as already stated, that Dr. Copland does not give the weight of his authority to a doctrine not only not proved, but altogether so improbable.

There still remain one or two topics connected with the history of Yellow fever that we had wished to notice; but we must pass them over altogether, and proceed, without further delay, to examine, and that very briefly, the Report of the Commissioners of the New York House of Assembly on the quarantine laws of that state. We had hoped to have been able to give a con-

* As a matter of course, whenever the pestilence does not appear for a number of successive years, the immunity is at once attributed by our author to the salutary effects of the quarantine which has kept the disease out. But to shew how fallacious such a mode of reasoning may be, it is worthy of notice that we have the high authority of Humboldt, in stating (we quote from Dr. Gillkrest) that, "although the disease usually exists among the newly arrived every year at Vera Cruz (one of the most pestilential spots in the world), it never prevailed epidemically there between 1776 and 1794, although the intercourse with the Havannah and other places, where the disease continued to prevail, was quite free. He even says that there was not a single example of the *vomito* during the eight years preceding 1794, although the concourse of Europeans and Mexicans from the interior had been very great."

densed analysis of the medical testimony in this Report; our want of space, however, prevents us doing so at present. As usual, there is no little discrepancy of opinion among the medical witnesses upon many of the questions that were proposed to them. The weight, however, of influential evidence is unquestionably in favour of the infectiousness and consequent importability of the disease; but not to the extent that Dr. Copland might lead his readers to suppose. Take the following as an instance. Dr. C. says;

"From 1751 until 1791, this pestilence made its appearance in New York on several occasions; but after the latter period it appeared more frequently and more destructively, as might have been expected from the increased size and population of the city, and the more frequent intercourse with places where it prevailed. It even occurred during two or three successive years, and was seldom absent for a longer period than this, until 1822, when it prevailed most fatally. Since that year quarantine regulations have been strictly enforced, and the distemper has not appeared again in that city—now a period of nearly a quarter of a century—although it has scarcely been a year absent from vessels detained in quarantine, and in the quarantine hospital.

"Can any stronger proof of the propriety of enforcing these regulations be adduced than that to which I have now referred?" Vol. III., p. 237.

To estimate, however, fairly the value of the statements here made and the conclusion thence deduced, the reader should be informed of one or two circumstances that cannot fail to qualify in some measure the opinion which he might otherwise at once adopt. The following extracts from the Report will explain what these circumstances are.

"The State of New-York, as early as 1784, enacted quarantine laws, and the examination of them will show that they are scarcely behind those now existing, in their practical operation; and yet in 1798, when the city contained only about fifty-eight thousand inhabitants, between two thousand four hundred and two thousand five hundred persons died with the disease the law was passed to prevent. At subsequent periods laws have been enacted, and precautionary measures taken, still, in the years 1805 and 1822, the city found itself in the midst of pestilence, and, in some other years, cases have occurred, but the number has been so small, that the disease could hardly be regarded as epidemic, and they have not been as numerous, if we except the years 1805 and 1822, as at the quarantine grounds." P. 41.

Moreover, we learn that there was no Yellow Fever in New York from 1805 to 1822, a period of *fourteen years;* therefore before "the quarantine regulations have been strictly enforced."*

---

* To give an idea of the excessive stringency of these regulations, we quote the first:

"All vessels direct from any place where yellow, bilious-malignant, or other pestilential or infectious fever existed at the time of their departure, or which shall have arrived at any place, and proceeded thence to New-York, or on board of which, during the voyage, any case of such fever shall have occurred, arriving between the thirty-first day of May and the first day of October, shall remain at quarantine, for at least thirty days after their arrival, and at least twenty days after their cargo shall have been discharged, and shall perform such further quarantine as the health officer shall prescribe." P. 51.

Thirty days quarantine imposed upon a vessel, although not a single case of fever has ever been on board!—and this too after it has been declared that "the yellow fever will develop itself in from two to twelve days after infection,

The exemption of the city from any invasion of the pestilence since 1822,* is attributed by our author, as well as by one or two of the medical gentlemen of New York, whose communications appear in the report, entirely to the more stringent operation of quarantine regulations; but then they omit to mention, not only that the general sanitary condition of that city has undergone a most material improvement since the period mentioned, by the removal of the crowded grave-yards, the purification of the quays, the cleansing of the streets, &c., but also that—and this is a very important circumstance—while a most rigorous system of defence and exclusion has been adopted, during the last twenty-five years, at New York against *suspected* as well as against *infected* vessels, an infinitely less severe code of regulations has been in force all the while at Brooklyn, which is only a few hundred yards distant, and between which and New York there is an incessant and unrestricted communication. We gather this from the following statements:

"Vessels arriving at the quarantine from interdicted ports with a healthy crew, not allowed to come to New York, have usually been permitted to come to the wharves at Brooklyn, after two days quarantine, and immediately discharge their cargoes, and the vessel remain at the wharves, or reload for any outward port." P. 17.

We are told by Mr. Brower, a most intelligent witness, that "after a vessel has discharged a healthy cargo at Brooklyn, during the quarantine season, and has been cleansed and ventilated, she is not permitted to come to New York to re-load until after the first of October, and these vessels are frequently thrown out of employ for months, while nothing can be more evident than if they were unhealthy at Brooklyn, it would be known as soon, and the effects as prejudicial to the health of New York as if they were at the wharves of the city. The contiguity and almost instantaneous communication between the two cities forbid any other conclusion."

It thus appears that there is good reason to believe that the health of New York during the last twenty-five years would, in all probability, not have suffered in the least, although its quarantine regulations had been much less rigorous than they have been all the while. We have alluded to the good effects of the improved sanitary condition of the city of New York, in rendering it less liable to the diffusion, if not to the invasion *ab extra*, of yellow fever. How strikingly is the truth of this exemplified in the following statement of Dr. Hort with respect to New Orleans, where he has resided for upwards of twenty years.

---

and even sooner, if the exposure is to the morbid effluvium of the disease in a concentrated form." It will be observed that no distinction is made between the (alleged) true or genuine pestilence and malignant bilious fever. Are we to infer from this, that they are regarded as one (or all but one) and the same disease? At all events, this circumstance alone shews how hard a thing it is to distinguish between what Dr. Copland and some other writers assert to be "essentially distinct diseases."

* "Since the year 1822 the city has been free from any epidemical yellow fever; yet some of our eminent physicians have expressed the opinion that deaths occur every year in the city, of a disease, if not the yellow fever, one which has all its characteristics except its epidemic, contagious, or malignant form." P. 14.

" Twenty years ago, only two streets, parallel with the river, were paved. In wet weather, the other streets were a perfect quagmire. In the back yards of the houses, there was only a thin crust of earth over an offensive black mud. At that time, most of the ground floors were almost in contact with the alluvial soil, and the back part of the city was low, presenting numerous pools of stagnant water. It was no wonder, under such circumstances, that a yellow fever atmosphere was generated. At present, the streets are paved. Yards are bricked, or flagged, or coated with asphaltum, which is also now generally used to coat the soil under the houses. The low parts, in the back of the city, have been filled up, and the wet swamp in the rear has been completely drained; all filth and offal in the streets is speedily removed, and the gutters are washed every day. There remains much to be done, however, before we can feel confident that a yellow fever atmosphere can no longer exist here. A great change has certainly taken place in the health of the city, which has been commensurate with the improvements carried on by the corporations and by individuals. Since 1841 we have had no yellow fever worth speaking of, and I feel assured that it is in our power, if the authorities will only adopt the necessary measures, to expel it for ever." P. 174.

The hope expressed here may be too sanguine; but the grounds suggesting it are at all events very interesting, and full of instruction. Dr. Hort is of opinion that the exemption of New York since 1822, and of Charlestown since 1832, is owing to " the general improvement in the cities, and greater attention to cleanliness particularly. It may also be in part owing to the changes of climate which we can observe, and to changes in the atmosphere which may happen, but which we cannot explain."

Several of the correspondents who strongly maintain that the Yellow Fever is an infectious disease, liable to be imported by shipping into New York, nevertheless declare it as their firm opinion, that the disease will not spread in places that are clear and well-ventilated. The Commissioners themselves use these words:

" If the yellow-fever, cholera, or small-pox are imported diseases, they may not be epidemic, except in a vitiated atmosphere, and if the atmosphere of the city is vitiated by bad practical sanitary regulations, then commerce should be relieved, and the city authorities or its citizens held responsible for furnishing disease with food to make it epidemic and destructive. Charging the epidemic yellow fever *solely* to commerce, guilty though she be in part, would be unjust, and laws to regulate her, be very inadequate to preserve the life of our citizens." P. 42.

The evidence of Mr. Allen, who was a member of the Board of Health during the epidemic visitations in 1819 and 1822, is strong upon this point. After stating his conviction that the disease was imported in these years by infected vessels coming from the West Indies, he says:

" It is a fact, too, that the disease, whenever it has prevailed in this city, has been confined to a particular district, and always commenced its ravages near the wharves of the city, while other parts, particularly the upper districts, have been exempt from contagion, and sick persons removed from the infected district to said parts, in no instance have communicated the disease to those attending them, or to others residing in their vicinity." P. 231.

What a striking illustration of this last remark is to be found in the evidence of Dr. Rodgers, who was health officer of New York, in the year 1805, when the yellow fever prevailed there in such a malignant form that no fewer than 270 out of 600 cases proved fatal.

" Of those attached to the health officer's department, boatmen, orderlies, and attendants, not one has been sick from any infection, or from any connection with the sick or infected vessels—of the lighters employed in carrying goods to the city or bringing cargoes to vessels at Quarantine, not one of them was, in the smallest degree, indisposed, till the 24th or 25th of September, when one of them was taken with fever, which left him in a few days; another was seized just after the first, and died on the 2d of October. Neither of them had any connection with any foul ship, to which they could attribute the complaint; but they took it from having had daily intercourse with the eastern part of the city, and from being under the necessity of going into houses and stores in that quarter, and staying longer in them than they had been accustomed to, by reason of the want of hands to receive their goods in consequence of the desertion of that part of the city." P. 11.

After quoting largely from Dr. Rodgers' official letter to the Board of Health, dated December 1805, the Commissioners add:—"This able physician and faithful health officer concludes his report by saying, I have now clearly shown, as far as negative proof can go, *that whatever might have been the cause of the late epidemic, it did not arise from any neglect of duty at the Quarantine ground,* NOR DID IT COME THROUGH THAT CHANNEL."

Equally strong is the testimony of Dr. Bayley, who was health officer during the epidemic of 1822. Of 70 persons sick with the fever, who were sent to the marine hospital on Staten island, 37 died, of whom 18 had black vomit. These cases were regularly attended by the physicians and nurses of the establishment, not one of whom was affected by the disease; "nor has a single case," says the doctor, "come to our knowledge of any person taking it, who was engaged in transporting the sick from the city to the hospital."

The inference, that surely may be drawn with confidence from these statements is that, admitting the general question of the infectiousness of the disease to be clearly made out, the risk of its spreading in a pure atmosphere is shewn to be exceedingly small. The case of Mr. Wells, related at page 10 of the Report, is an apt illustration of this. It would be easy to quote a host of others to the same effect.

We must not quit the examination of the Report before us without directing especial attention to the condition of Boston in reference to the question as to the importation of the Yellow fever, and the proper measures for its prevention and arrest. Boston has been singularly exempt from any severe visitation of the disease. It appeared there in 1798, in 1802, and again in 1819; but on none of those occasions did it spread to any extent, or cause great mortality. Now it seems that there is no fixed or standing code of quarantine regulations in that port against yellow fever or any other febrile disease in particular; at least, nothing at all approaching to that in force at New York, of which we have given a specimen. In place of there being any specified number of days appointed for the isolation of a merely-suspected vessel coming from a particular port, the harbour physician—who (a not unimportant circumstance) is paid by a fixed salary, and not by fees payable by the masters of the vessels—is invested with ample discretionary, but responsible, powers to adopt all means that he may deem proper to prevent the development or introduction of any infectious malady, and arrest its diffusion. The following two regulations embody his chief duties:

" It shall be the duty of the said Physician to examine into all nuisances, sources of filth, and causes of sickness, which may be on board of any vessel within the harbour of Boston, or which may have been landed from any vessel, or any wharf or other place; and under the direction of the mayor and alderman, to cause the same to be removed or destroyed." P. 237.

" Whenever any vessel shall arrive in the harbour of Boston, which is foul and infected, or whose cargo is foul and infected with any malignant or contagious disease, or any of whose crew or passengers are sick with any malignant or contagious disease, it shall be the duty of the port physician forthwith to report the same to the mayor and aldermen, and if directed by them, to cause the said vessel, her cargo and crew, or either or any part thereof, to be removed to the anchorage ground or wharf at Rainsford island, and the said vessel and cargo to be thoroughly cleansed and purified at the expense and charge of the owners, consignees or possessors of the same. And also, when directed as aforesaid, to cause all or any persons arriving in such vessel, who are sick of any malignant or contagious disease, to be removed to the hospital, on the said island, and all expenses incurred on account of such persons shall be paid by themselves."* P. 238.

By acting upon this simple and intelligible system, we are informed that " the city (of Boston) has been protected, for many years, from the introduction of any malignant or infectious diseases."

In the evidence respecting Boston, as well as that respecting New York, allusion is made, on more occasions than one, to the Yellow fever having made its appearance on board vessels when their cargoes have been begun to be moved or their holds exposed, although the crew had remained healthy during the voyage, and were so at the time of the ship's arrival in port. In illustration of this point, we give the following instance: it is mentioned in the evidence of Dr. Sweetser.

" In July, 1819, the ship Ten Brothers arrived at Boston from the coast of Africa. Her crew were all in good health and had been during her voyage. She remained a fortnight at quarantine, and then came up to the wharf, all on board continuing well. Her hatches were now removed, when immediately a large part of her crew, and also various others who had been employed on board,

---

* The Boston code of quarantine regulation is in accordance with (was it suggested by?) the sentiments of a very able and enlightened writer in the 10th volume of the North American Review. The following passage appears to us to be replete with the best practical wisdom; it contains, we think, the key of all sound legislation upon quarantine matters.

" The application of quarantine laws ought not to be regulated in reference to the places from which vessels arrive, *but by the state in which they arrive.* There is no cause for detaining a ship on account of the danger of yellow fever, which is itself in a pure and healthful state, from whatever port she may have sailed, nor however sickly that place may have been. On the other hand, no ship that is foul and offensive, or that has a cargo in a putrid state, although the place from which she sailed, or the persons on board be ever so free from sickness, ought to be permitted to approach the town until she has been thoroughly cleansed. There ought to be a rigorous system of inspection, during the summer and autumn, into the state of every ship which has a cargo of a perishable nature, while she is discharging. In this manner the danger, when it exists, will be detected before it can have extended to any considerable number of persons, and will be promptly removed." P. 46.

as custom house officers and labourers, and even the crews of other vessels lying alongside, were seized with yellow fever, and which, in most of the cases, terminated fatally. The hold of this vessel had become exceedingly foul from putrefying corn. At this time, 1819, so entirely was the system of quarantine founded on the assumption of imported contagion, that the internal condition of the ship itself was not at all regarded, hence the Ten Brothers was not even subjected to ventilation at quarantine, her hatches not even being removed till she reached the wharf, when there issued from her close and foul hold such concentrated streams of poisonous effluvia, that nearly all who were exposed to them perished of a malignant yellow fever. That yellow fever, however, be thus produced, the aid of certain incidental influences would seem to be demanded, since foul ships are constantly arriving in our ports, while it is only occasionally that this fever is generated by them. A long-continued elevation of temperature, united with stillness of the atmosphere, are among the adjuvant circumstances conducive to the development and activity of the specific poison originating yellow fever." P. 158.

If the Report of the New York Commissioners is favourable to the opinion of the Yellow fever being, under certain circumstances, infectious and liable to be introduced into healthy parts by vessels having the disease on board, that of Dr. Mc William on the Boa Vista epidemic must be deemed conclusive. We earnestly solicit the attention of our readers to the analysis which we are about to give of this very able document; as, from the strict accuracy of its details, and the great care with which it has been prepared, it cannot fail to be appealed to, not only by all future professional writers on the history of Yellow fever, but also by the Legislatures of this and of other countries whenever the subject of quarantine in connexion with this disease is brought under their consideration, Being a parliamentary paper, and therefore not likely to fall into the hands of many medical men, our summary of its contents shall be as complete as possible ; the main wish and object which we have had throughout this article being to bring forward a mass of admitted facts and statements, so that the reader may be enabled to form his own judgment of their value, apart from any reasonings of ours.

In the number of this Journal for July of last year, we gave a minute account of the very fatal fever which prevailed on board the "Eclair," from the time of her leaving the African coast until she arrived in England. It was then stated that, in consequence of news having come to this country, that the disease had broke out with great malignancy at Boa Vista soon after the steamer's departure, the Admiralty had sent out Dr. Mc William,—than whom, from his talents and experience, a better selection could not have been made—to that island, with the view of ascertaining all the particulars of the case, and more especially of determining, as accurately as possible, the point whether the fever raging there had been introduced by the Eclair, or was of local and endemic origin.

At the close of that article, we gave it as our opinion, after a candid examination of all the facts that were at that time known, that the disease "had been imported by the infected steamer into Boa Vista." It will be seen presently whether that opinion was correct or not; but, before entering upon details, it may be well very briefly to recapitulate the leading events in the outbreak and spread of the fever on board that vessel, before and after her arrival at the Cape de Verd islands.

The " Eclair" left England for the coast of Africa in Nov. 1844. She reached Fernando Po on the 25th of December. For two months, she kept cruising along the coast. On the 23rd of February, she was at Sierra Leone, and took on board a complement of forty Kroomen. After remaining there for a few days, she returned to Seabar, off the island of Sherboro. At this place, the men were much engaged in the boats, exposed to the heat of the day, and to the dews and tainted atmosphere of the night, in that unwholesome climate. The water also, which they had for drink, was often foul and unwholesome. The first case of fever (remittent) occurred on the 3rd of April: the man recovered. The next three cases proved fatal. No fresh cases occurred from the 18th of April to the 22nd of May, when three more of the men were attacked; all died, one on the 5th, another on the 7th, and the third on the 13th day. During the next fortnight, other five cases occurred: four of the patients died in from four to nine days. The fever after this seems to have ceased for several weeks; *i. e.* from the 8th of June to the 19th of July, when the Eclair again reached Sierra Leone. While there, the men were engaged in cleaning out the Albert steamer which was in a most foul and offensive state, and, while employed in this most unwholesome duty, had unfortunately the opportunity of indulging to excess in the use of ardent spirits. On the day last mentioned, the fever re-appeared on board the Eclair: the man died on the eighth day. During the next three days, three fresh cases occurred: all proved fatal. On the 23rd, the Eclair left Sierra Leone, having the Albert in tow: they proceeded to the northward. Three fatal cases of fever occurred within a few days after sailing. A merchant, who had embarked in the Albert at Sierra Leone, died on board that vessel on the 27th. On the 1st of August, other two cases occurred: the patients recovered. On the 2nd, there were two fresh cases: they proved fatal. On the 8th and 9th, four men were added to the sick list: two died, and two recovered. On the 10th, the steamers reached the Gambia. After remaining till the 14th, the Eclair sailed for Goree, having four sick of the fever on board. On arriving there, she was refused pratique: one man died there. On the 17th, she proceeded to Boa Vista, which she reached on the 20th: three men had died on the passage. During the first ten days she was at Boa Vista, five fatal cases occurred. The men, the healthy as well as the sick, were then landed on the small island at the entrance of the harbour, and the vessel was overhauled: the officers, &c. were accommodated at Porto Sal Rey, the chief town of Boa Vista. Notwithstanding the removal of the sick on shore, the fever continued to prevail, and even to increase; for no fewer than 28 deaths took place between the 31st of August (the day of landing) and the 12th of September, when the Eclair sailed: making in all 39 deaths from the time of leaving Sierra Leone. When the Eclair was at Boa Vista, the " Growler" steamer arrived from Sierra Leone on the 6th of September, just as the assistant-surgeon of the former was taken ill. It was then that Dr. Maclure—who, having just received his promotion, was returning to England in the Growler—nobly volunteered his services on board of the Eclair; a memorable, but not a rare we trust, instance of heroic self-devotion! We need not pursue the rest of the sad tale of the vessel from the time that she left Boa Vista until she arrived in England; as it is altogether with the events, which occurred in the former place after her departure, that we have now to do. Before taking up the narrative of these, let us here premise that, from all accounts, the island was entirely free from sickness at the date of the steamer's arrival, as well as during her stay there. It is said by Mr. Macaulay to be " a remarkably healthy place," and to be by no means subject to any pernicious fevers. In some seasons, it appears that there is a good deal of remittent fever; and this has been known occasionally to assume a more than usually severe and fatal character. The last year when such was the case was 1833. In order that nothing at all important may be omitted in our narrative, we may state that the English consul, in his letter of the 22nd Dec. 1845 to Lord Aberdeen, alludes to the extraordinary heat and unusually heavy rains—" events which were surprising to the oldest inhabitant"—that had occurred up to the 9th of October.

Porto Sal Rey is, as we have already said, the chief town of the island: one of its districts is called Pao de Varella. Rabil is about four miles distant from Porto Sal Rey, and Moradinha about half a mile from Rabil.

While the Eclair was at Boa Vista, " the seamen seemed to have resorted chiefly to the house of a man called Justinian da Silva Georgio, who keeps a spirit-store in Porto Sal Rey. It is remarkable that this man was attacked with headache and general fever on the evening of the day he was visited by the Eclair's people; he was attended by Dr. Almeida and seen by many of his friends during his illness, which lasted a fortnight; amongst the latter were two females, called Anna Gaspar and Rosinha San Antao, both of whom had slight fever shortly afterwards. It is but necessary to mention that both these women (prostitutes, we suppose.—*Rev.*) had been visited by people from the Eclair; they soon recovered, and their illness at the time attracted no notice whatever."

These were the only cases of sickness that occurred among the inhabitants of Porto Sal Rey, during the stay of the Eclair at Boa Vista. It should be also mentioned that, while the officers &c. resided on shore, several of them, the captain's cook, and some other servants were taken ill, and were, according to the strict orders of Capt. Estcourt, immediately conveyed to the fort at the entrance of the harbour.

Let us now see what occurred to the small military guard stationed there during, and immediately after, its occupancy by the crew of the Eclair. Dr. McWilliam's account is as follows:

" When the crew took possession of the Fort they found a guard of three soldiers there. They had been on this post already three or four days. Their names were—

Athanasio Perez, Acting Corporal, dark mulatto, aged 26 years;
Pedro Manoel, private, negro, aged 30;
Antonio dos Santos, private, mulatto, aged 25.

This guard slept in front of the upper room occupied by the sick, and often went into it. The corporal had a headache two days after the sick were landed, which at first yielded to cooling fomentations. In two days more he and his comrades were relieved, and he was still rather unwell, but making no complaint was posted as a sentry at the barracks in Porto Sal Rey. He was however soon taken ill, and sent to his house in Pao de Varella, where he was confined some weeks. His case caused no alarm, nor was he attended by any medical man, further than being seen once by Dr. Almeida during his convalescence. The other two soldiers had also attacks of fever, the one three weeks or so after he left the Fort, and the other not until fever was general in the barracks.

"The second guard at the Fort consisted of—

Vicente da Cruz Silva, dark mulatto native, aged 29;
Manoel Antonio Alves, negro native, private, aged 18;
Luis Briza, European Portuguese, private, aged 18.

"The guard was on duty at the Fort six or seven days; none of them were at all affected during this time. They however all had fever afterwards, when the disease was prevalent in the town, of which Luis Briza, the European, died.

"Third guard. In the routine of duty the second guard was relieved by

Corporal Joaquim Agostinho, European Portuguese;
Private Joao Alexandre Roque, European Portuguese; and
Private Miguel Barbosa, native negro, aged 30.

"By the evidence of Miguel Barbosa, the only survivor of this guard, it appears that he and his comrades were at the Fort several days before and after its evacuation by the sick of the 'Eclair.' They slept in a small cook-house under the rampart. The same day the 'Eclair' left, Miguel was ordered by the corporal Agostinho to sweep out the rooms which had been occupied by the sick, and that all three of them went into the rooms on this occasion. According to his account, on the day after the steamer sailed, the corporal was attacked with fever and died in three days, delirious, and vomiting a black fluid. The other European, Joao Alexandre Roque, was similarly seized on the day following, and died on the fourth day with fever, delirium and black vomit. Both these men were seen by Dr. Almeida, who told me they were in *articulo mortis* when he was called to them.

"Pedro Manoel, a native negro soldier, was sent by Major Mascarenhas, the Military Commandant, to attend the sick soldiers at the Fort; he was with them from an early period of their illness up to the time of their death, and remained at the Fort for several days afterwards, in company with Miguel Barbosa. Before they left the fort some gunpowder was exploded in the rooms which the 'Eclair's' people had occupied; and it is said that they were whitewashed some time afterwards.

"Another negro soldier, called Manoel Antonio Alves, who was on duty with the second guard, was ordered by the Commandant to bury the bodies of Agostinho and Roque. For this purpose he proceeded to the small island accompanied with Joaquim Farenga and Manoel Luis, both native soldiers. He left his comrades in the boat, and stripping himself, ran up to the Fort in a state of nudity, and there, with the assistance of Miguel Barbosa and Pedro Manoel, rolled the bodies in quilts, and carried them down to the boat, which transported them to the beach, about a mile and a half to the southward of the town, where they were buried in the sand.

"The fourth guard was constituted of this man, (Manoel Antonio Alves,) Lorenzo Samed, and Pedro Gonzalves, all native soldiers. The same boat, that conveyed them to the Fort, on its return brought Miguel Barbosa and Pedro Manoel to Porto Sal Rey. The Commandant, however, deemed it advisable not to admit them at once into the barracks, but sent them to a house in the upper and northern end of the town called Beira,* which is a part of the District Pao de Varella.

---

* "This is a row of four small houses, divided from each other by rough rouble partitions. The uppermost was occupied by a mulatto native woman of the name of Theresa Maria Jesus; the next by the two soldiers; the next to that by a Portuguese woman called Anna Gallinha, and a native woman named Anna Texeira; and the other by a Portuguese man called Jose Carlos da Lisboa, a writer."

"The soldiers of the fourth guard lived in a room next to that in which the sick of the 'Eclair' had been. Manoel Antonio Alves was taken ill in the course of two days, and, strange to say, was at once conveyed to the barracks in Porto Sal Rey. Pedro Gonzalves and Lorenzo Samed were in their turn in the course of ten or twelve days relieved from duty at the Fort. Gonzalves was taken ill with fever some days after he returned to the barracks, and died on the 26th of November. Lorenzo Samed was also attacked, and recovered after a dangerous illness.

"The fifth guard at the Fort, Andre Vass and Jose Sancha, two negro private soldiers, were sent to the Fort to replace the fourth guard. Jose Sancha was attacked with fever three days after he had been there; upon which the Commandant resolved for a while to withdraw the guard altogether from that fatal post. Both the soldiers therefore returned to the barracks, where Vass was also taken ill in the course of three days." P. 84.

From this statement it appears that the only fatal cases among the guard at the Fort were those of the two Europeans, who were there when the sick of the Eclair left on the 12th of September. Both of these men sickened a day or two afterwards, and died within the next three or four days; *i. e.* about the 20th of the month or so. Some, indeed, of the other soldiers also—who, it should be remembered, were either mulattoes or negroes—had been ill; but only in a slight degree.

Let us now follow the two men Barbosa and Manoel, when, upon leaving the Fort about the 25th September, they went on shore to live at Beira. "It is of the utmost importance," says Dr. Mc William, "that every circumstance connected with their stay here be carefully noted, as it afterwards turned out that the very first (fatal) case of fever in the town appeared in the room adjoining that in which these two soldiers were lodged." Although neither of them was so ill at this time as to be confined to bed, it is obvious that they had the germs of the disease about them.* After remaining in the house for a week or so, they went to the barracks where, it seems, both were laid up. Among their most frequent visitors at Beira were their neighbours Gallinha and Texeira; the former, indeed, had been in constant attendance upon them, and had cooked their provisions. She was attacked about three or four days after they left, and died with high fever, delirium, and black vomit. This was on or about the 16th of October—a little more than a month after the sailing of the Eclair. Up to this time, no apprehension had existed among the residents of Porto Sal Rey, although it was known that two soldiers had previously died in the fort, some weeks before; it was then thought that the disease would not spread to the town.

---

* "Their clothes were taken by the wife of Silvester Jose Romess, a negro mason, to be washed. Silvester himself had also been a visitor of the soldiers when in the house at Beira; he had never been on board the 'Eclair' or at the Fort, but was attacked with fever soon after the soldiers returned to the barracks, as were also his daughter, niece, and wife, but the latter not until the fever had become general in the town."

It may be as well to mention here, that of 17 women who washed the soiled linen brought from the Eclair, not one of them was seized with the fever before the end of October, by which time the disease already prevailed in the town; "so that in none of these cases can the occurrence of the fever be fairly attributed to infectious matter conveyed by the linen."

The next fatal case occurred in a mulatto man, Affonso by name, who lived only twenty yards from Anna Gallinha, and who had frequently visited her; he died two days after her. A native mulatto woman, who lived next door to this man, and who visited both him and Anna Gallinha, was attacked with fever on the 19th: she also died. The man, who carried the bodies of Affonso and of Gallinha to burial, was two days afterwards seized; he died. The persons who had waited upon him, straightway sickened: one of them died. The occupants of the other two houses in the Beira were taken ill about the same time. Theresa Jesus, and also Anna Texeira (who lived in the same room with Anna Gallinha) recovered; but Lisboa the writer, a Portuguese, died on the 27th or 28th. "Among those who visited this person, was the son of Senhor Carvahal, merchant in Porto Sal Rey; this youtn, aged 18, had passed two nights in the house of his friend. He sickened the very day that Lisboa died, and died himself on the 4th November. He had not, as far as could be discovered, had any intercourse with any other infected person, save his friend. He was nursed by his mother, sister, and father, who, like himself, were dark mulatto natives; but none of them suffered.

"The disease had attacked several persons in Porto Sal Rey proper, or the lower part of the town, by the end of October; and some of the soldiers were sick in the barracks at this time. The force on the island is in general about forty soldiers, who, with few exceptions, are natives of the Cape de Verds. Before the late invasion of fever, there was one major and commandant, one lieutenant, and forty-one soldiers, including serjeants and corporals. Of the forty-one soldiers, thirty-two were negroes or very dark mulattoes, and nine were European Portuguese. The whole were attacked with fever, which proved fatal to eight of the nine Europeans; two of them died at the Fort, and the other six in the barracks at Porto Sal Rey. Of the native soldiers three died." P. 86.

Meanwhile, the Governor with his suite, several of the better class of Portuguese, and the greater number of the English, had left the island about the end of October for other islands of the group, where rigid quarantine was kept up against Boa Vista. "The whole of those," says Dr. M. "who left the island, have, I believe, continued perfectly free from fever, with the very notable exception of one family which returned to it in November," under the belief that the disease had nearly, if not altogether, disappeared. The particulars of this case are too interesting to be passed over:

"Mr. Pettingal and his family, consisting of his wife and daughter, a young lady about twenty years of age, were lodged for the first night on their return to Boa Vista, in the house of Mr. Macaulay, and on the following day removed to their own house. A day or two subsequently, two mulatto girls who, during the absence of the family at San Nicholao, had been in charge of the house, were both taken ill with fever; and although one was sent home immediately, the other remained in the house three days after she was attacked, and was during this time frequently visited by Miss Pettingal. Both of the mulatto women recovered; but on the 17th of November, or six days after the return of the family to Boa Vista, Miss Pettingal was seized with symptoms of fever, which in forty-eight hours were unequivocal, being marked by black vomit and retention of urine, and terminated fatally on the afternoon of the 24th November, or the seventh day from being attacked.

"Miss Pettingal was sedulously attended during her illness by Mrs. Learner, an English nurse, and constantly visited by her father, mother, and Dr. Kenny, her medical attendant. The captain of an American merchant-ship, who had been at Porto Sal Rey for some weeks, very frequently visited Mr. Pettingal's house during his daughter's illness, and assisted, at least on one occasion, in shifting the bed on which she lay from one room to another. John Dachin, an English servant of Mr. Macaulay, also assisted during Miss Pettingal's illness, and slept one night in the house.

"John Dachin was attacked on the 25th November, and died on the fith day with the most malignant symptoms of yellow fever in the house of Mr. Macaulay. Mrs. Learner must have been attacked almost immediately after Miss Pettingall's death, for she died on the 27th November.

"Dr. Kenny was taken ill about the same time, and also died on the 27th, at Rabil." P. 87.

The American Captain afterwards sickened and died; and subsequently Mr. Pettingal himself fell a victim in the first week of December.*

During the latter half of November, and throughout December (1845), and part of January (1846), the disease was at its height in Porto Sal Rey; sometimes six or seven persons dying in one day.

The fever in the town began to assume a milder character in the month of February. "There were a few cases in March, and I saw," says Dr. M'William, "three so late as early in April. In these, there was saffron-coloured suffusion of the eyes, but no black vomit. Two of them were blacks, and the other was a European boy. All recovered. By the end of April, the town was quite free from fever."

The following is a tabular view of the mortality which occurred in Porto Sal Rey:

EUROPEANS.—Portuguese—

| | |
|---|---|
| Number exposed to fever .. .. .. | 53 |
| " attacked with fever.. .. .. | 47 |
| " died .. .. .. .. | 25 |

Ratio of deaths in the population, 1 in 2·1
Ratio of deaths in number attacked, 1 in 1·8

| | |
|---|---|
| English, including two Americans who were exposed to the fever .. .. .. | 11 |

* In one of the northern villages of the island, a case somewhat similar to that of the Pettingal family occurred. Of 23 persons, who left a place where the fever existed, 8 returned some time after, while the disease however still prevailed in the village. All these caught the fever, and 4 of them died. The remaining 15, who did not return, entirely escaped.

"The exemption," says Dr. M'William, "in persons who removed in time from infected places was clearly shown in many instances. Dr. Almeida, by changing the residence of his family from place to place, succeeded in keeping the whole of them intact. The same gentleman for some time prevented the introduction of the disease into Fundo das Figueiras, by the establishment of a sanitary cordon; and afterwards retarded its progress by the imperfect means of segregation of the sick which he had in his power. Boaventura was for some weeks without a case of fever, although the disease was raging at Cabeçada, a few hundred yards only from it, by the adoption of measures to prevent intercourse."

| | |
|---|---|
| Number attacked .. .. .. .. | 8 |
| „ died .. .. .. .. | 7 |
| Ratio of deaths in population, 1 in 1·6 | |
| Ratio of deaths in number attacked, 1 in 1·1. | |
| French— | |
| Number exposed to fever .. .. .. | 2 |
| „ attacked .. .. .. .. | 2 |
| Spaniards.. .. .. .. | 2 not attacked |
| NATIVE POPULATION.—Free .. .. .. .. | 666 |
| Slaves .. .. .. .. | 249 |
| Total .. .. | 915 |
| Died, 65 free, 3 slaves .. .. | 68 |
| Ratio of deaths in native population, 1 in 13·4 | |

So much for the outbreak and course of the fever in Porto Sal Rey. The reader must now have his attention directed to what was going on, in a very early part of its career, at the town or village of Rabil, which is divided into two districts, known by the names Cabeçada and Boaventura. Here it is necessary to state that of the men, upwards of 40 in number, who were employed on board the Eclair during her stay at Boa Vista, and all or most of whom returned every night to their homes, not one appears to have suffered either before or for a considerable time after the departure of the steamer, save one man from Cabeçada, Luis Pathi, whose case is thus given by Dr. Mc William :

" He returned to his house when the vessel sailed, and on the following day went to Moradinha (or Santa Cruz), a hamlet on the south-east side of the Rabil ravine, to attend the feast of the Exaltation of the Holy Cross. This festival is observed on the 14th of September, and its celebration sometimes continues three or four days longer. While engaged in the dance on the third or fourth day of the feast, he was suddenly taken ill, and at once put to bed, where he remained several days; at the end of this time he was conveyed to his own house in Cabeçada, where he was confined three weeks longer. He had general fever, pain of back, &c., but no black vomit. His daughter, a girl of twelve years of age, was attacked on the tenth or eleventh day after his return to Cabeçada, and died in three days, with suppression of urine and black vomit. Another daughter, seven years old, was next seized, and died in four days; a boy eleven years of age was next taken ill, and died on the fifth day with symptoms similar to those of his sisters. His wife was attacked the same day the boy died; the disease in her case was protracted to fifteen days' duration, and was marked in its last stage, as it had been in the cases of her children, by black vomit.

" Such was the ominous commencement of the fever in the populous district, Cabeçada."* P. 89.

---

* It thus seems that the fatal case of Pathi's daughter occurred some days before that of Anna Gallinha in Porto Sal Rey. Here it may be right to state that although Pathi, as we have seen, sickened at Moradinha, and remained there in bed for several days before returning to his own house at Cabeçada, yet no other case of fever occurred in the former place until the month of December, when a woman came sick from Rabil, and died shortly afterwards.

It is scarcely necessary to follow the course of the pestilence in this place. All the evidence goes to shew that it originated from the family of Pathi. His house was situated in the most crowded part of Cabeçada, and adjoining to it were the houses of Manoel Fachina, Joaquim Marques, Joaquim Pathi, and Manoel Rosa, who, after Luis Pathi's illness, were the first sufferers in Rabil. The disease soon appeared in the other parts of the town, more especially in the south-western direction.

The fever was most fatal in Cabeçada in the months of November and December, and in Boaventura from the middle of November to January. Some cases appeared in February and March. Dr. M. saw two as late as the beginning of April; but the disease was then in a mild form.

We shall not pursue the outbreak and spread of the fever in the other villages on the island. Suffice it to say that, in Dr. McWilliam's opinion, " the disease could in each case be clearly traced to one or two individuals coming from an infected district, and affecting first the inmates of the house they remained at, next the visitors, and gradually extending on the whole of the inhabitants. In fact, it may be said that, in each town and village on the island, the disease first appeared in a single house, which became an irradiating focus for its dispersion in all quarters."

The fever was supposed to have ceased entirely throughout the island by the end of April; but, three weeks or so afterwards, it again made its appearance at Moradinha, the village where Luis Pathi was taken ill, but in which the mortality had been remarkably small during the prevalence of the epidemic. Dr. McWilliam at once proceeded to the spot, and he found two persons, a girl of 14 years of age and a man about 35, with all the symptoms of a most malignant fever. His description of these symptoms is so vivid that we must not fail to give it:

" The countenance of the girl, a dark mulatto, was of a dirty lemon colour, shining through the natural darkness of the skin, made it resemble very much that of a light bronzed statue. A very strong fœtor issued from the body, which tainted the room and drove us back from the door until the window was opened. She had complained much of pain along the spine, and still pointed to her head as the seat of pain. She had been bled in the arm by one of the neighbours, and all around the wound was of a greenish colour, swollen, and putrid. In the angles of the mouth there was dark frothy blood. She had had black vomiting; but this symptom had for some hours ceased. The urine was black, as were also the fæces. The former had been voided in very small quantities. Pulse small, irregular. She had been ill seven or eight days.

" The man had nearly the same symptoms, but in a milder degree, and he had not been affected with black vomit." P. 94.

The girl died next day; the man gradually recovered. He appeared to have been benefited by the use of small and repeated doses of Quinine. Dr. M. learned that there had been two other fatal cases of the fever in Moradinha, some days before it was discovered that the disease existed there. Four fresh cases occurred between the 1st and 3rd of June. " In none of them," says Dr. M., and we particularly invite the reader's attention to this remark, " was there black vomit; they soon passed from the *continued* to the *remittent* form, and all recovered under the use of quinine, mild aperients, and nourishing diet."

Nevertheless, it is but right to mention that Dr. M. has remarked, in his concluding observations, that "remittents and intermittents were much less common among the convalescent than I had expected."

The account, given of the re-appearance of the fever at Moradinha, is as follows:

"A girl called Maria dos Prazeres was the first person attacked at Moradinha on this occasion. She had visited Joao Gallego and the other eastern villages on the 15th of May, and returned to Moradinha where she was laid up with fever on the 20th of the same month. Her mother who slept with her one night after she was taken ill, was also attacked on the 26th and died on the 29th, after three days' illness. The girl whom we saw was called Perpetua. She had visited Maria, soon after which she was seized with fever, and died with the symptoms which have been already described, on the morning of the 1st of June.

"The disease did not extend to Rabil or any of the other villages, and on the 13th of June the whole of the remaining patients were convalescent; and when I left the island on the 15th of July last, there had been no case of fever for thirty-three days, and no deaths had occurred since the 1st of June, or during a period of forty-five days." P. 95.

The following table shews the total amount of the actual and of the relative mortality among the different classes of the inhabitants of the island of Boa Vista, during the prevalence of the fever.

| | Total Resident Population. | Left the Island. | Exposed to Fever. | Died. |
|---|---|---|---|---|
| Europeans :— | | | | |
| Portuguese .. .. .. | 56 | 3 | 53 | 25 |
| English and Americans | 25 | 14 | 11 | 7 |
| French.. .. .. .. | 2 | .. | 2 | .. |
| Spaniards .. .. .. | 3 | .. | 3 | .. |
| Total Europeans .. | 86 | 17 | 69 | 32 |
| Natives :— | | | | |
| Free 3875.. .. .. | 3875 | .. | 3875 | 266 |
| Slaves 434.. .. .. | 434 | .. | 434 | 13 |
| Total Numbers .. | 4309 | .. | 4309 | 279* |
| Grand Total .. .. | 4395 | 17 | 4378 | 311 |

*Ratio of Mortality*

| | | |
|---|---|---|
| Amongst Portuguese, Spaniards, and French, who were exposed to the Fever | .. .. .. | 1 in 2·28 |
| " English and Americans | .. .. .. | 1 in 1·60 |
| " Native population—Slaves | .. .. .. | 1 in 33·4 |
| " Free | .. .. .. | 1 in 14·6 |
| Average mortality of native population | .. .. .. | 1 in 15·4 |

* To this number we must add 3 more out of the eight additional cases, which occurred at Moradinha between the 20th of May and the 3rd of June.

Dr. McWilliam found it quite impossible to ascertain, with any degree of correctness, the number of persons attacked in the several villages; but, from the best information he could obtain, he believed that about two-thirds of the whole population of the island was affected by the fever.

When he left the island at the end of the second week in July, no case of fever, as we have seen, had occurred for upwards of four weeks, and no death for six weeks. The disease had not, in any one instance, extended to any of the other islands of the Cape de Verd group. A rigorous quarantine had been kept up, during the whole time, against Boa Vista. It was stated in Dr. Stewart's report that yellow fever existed at Porto Praya in San Jago, while the 'Eclair' was at Boa Vista. This statement, we are told by Dr. McWilliam, is not correct. The only fever at that time existing in San Jago was the usual endemic remittent, which prevails there every season during and after the rains; nor was it then more severe than ordinary.

There cannot be a doubt but that the fatality of the epidemic at Boa Vista was not a little aggravated by the great scarcity of provisions which had existed in the island for some months, and by the utter want of any medical assistance whatsoever during the greater part of the prevalence of the fever. The poverty and the destitution of the people, the filthy state of the streets and of the houses, the presence of a quantity of stagnant putrid water in the immediate vicinity of the town of Porto Sal Rey, in conjunction too with the very great heat of the weather, must all have contributed to render the febrific poison more fatally malignant.

The mortality occurred chiefly among persons of from 12 to 35 years of age, and death took place generally between the third and seventh days.

"Of second attacks," says Dr. M., "I could only find five cases at all clearly established; from the little appreciation that the people have of time, it is not possible to arrive at the exact intervals between the attacks."

Let us now see what are the general deductions, which the conscientious and enlightened reporter has drawn from a calm and minute examination of all the facts connected with the memorable history of the "Eclair fever" throughout its entire course.

"Under ordinary circumstances, the fever which attacks ships' crews on the African coast is the common bilious remittent. Seldom if ever has it happened that a disease characterized by black vomit has at once broken out in a ship. This malignant symptom rarely occurs, even on shore, except during some of the more severe epidemic visitations. Judging, therefore, from the result of general experience, as well as from the medical reports, it seems to me to admit of no doubt that the disease which invaded the 'Eclair' in the latter end of May and early part of June 1845, was the usual endemic fever of the African coast, which although a most fatal disease, is not considered to be of an infectious or contagious nature. The mental despondency which seems to have pervaded the crew rendered them indeed peculiarly susceptible of disease, not only while on detached service in the boats up the river, but also on board the ship (around which the commonly recognized causes of fever were in great abundance), and on this account it is probable that the fever was of a more aggravated type than usual.

"At Sierra Leone, the irksome and unwholesome duty of cleaning the hold of the 'Albert,' as well as that of their own vessel; the unwonted exposure in some cases by day and night; the irregularities committed by men who had for some months been exposed to morbific influences; all combined to the development of a fever of a much more malignant nature than the usual endemic of the African rivers.

"Accordingly, several of the cases that occurred after the ship left Sierra Leone were marked by unequivocal black vomit—a symptom, as has been already mentioned, extremely rare in the endemic fever, and regarded by all who have served in hot climates, as a test of unusual malignancy.

"It is thus quite evident that the type of the fever changed materially for the worse during the passage from Sierra Leone to the Gambia and Boa Vista. There is also great reason to believe that it acquired still greater virulence while the crew were at the Fort. The house in which the sick were lodged there contains only one room at all well ventilated; and, judging from the evidence of Dr. Almeida,* they must have been much crowded; at all events, the fact is beyond doubt, that the accession to the sick list and the mortality became much greater at this than they had been at any previous period. In short, from the endemic remittent of the African coast, the disease had, from a series of causes, been exalted to a concentrated remittent, or yellow fever." P. 105.

Such is Dr. McWilliam's deliberate opinion; an opinion, it will be perceived, in entire accordance with the whole of the reasonings and statements adduced in the early part of this article, which, it is but fair to ourselves to state, was written some time ere we had received Dr. M.'s report. The following passage cannot fail to be read with interest, as an interesting comment on much that has gone before.

"Sir William Pym and those who espouse his doctrines will contend that the fever of the 'Eclair' was from the first a contagious disorder, differing essentially from the remittent of the African coast. If a disease such as that described by Sir William Pym be really endemic on the coast, surely we ought to hear more of it, considering the large squadron which is now kept there.

"If it be assumed that the fever on board the 'Eclair' was the Bulam, and therefore primarily contagious, I would ask where was it contracted? Not at Seabar, for in that case we would expect other vessels that visited that part of the station to be affected with a similar disease. Was it at Sierra Leone? For if so, why were not some of the many Europeans from the numerous shipping there, attacked with a fever manifesting contagious qualities? And we know that while the 'Eclair' was there, there was nothing unusual either in the amount or nature of disease in the colony. The same may be said of the Gambia. Assuredly the remittent is quite destructive enough of human life; and in the late Niger Expedition, where its fatal effects were manifested in a terrible degree, there was no reason to believe that its spread was due to contagion. The whole of the surviving sick were landed at Fernando Po, after we got clear of the Niger, yet none of the residents there suffered in consequence, although many officers and men died at Clarence Cove. Why did this malignant disorder rage on board the 'Eclair' and not in other vessels that were with her? Simply because her circumstances were peculiar, and it is entirely to this peculiarity and unwonted

---

* "You were, I understand, requested to see the people of the 'Eclair?'—Yes, I was requested by the Governor to go to the Fort and see the sick of the 'Eclair' there.

"How did they seem to be lodged there?—They seemed to be extremely crowded. I could hardly pass between them. In one small room there were about twelve sick persons.

"How many sick were there altogether when you visited them at the Fort?—I should say about thirty, if not more."

combination of circumstances that the contagionality of the fever with which her crew was affected is due. To me there is no proof that the fever in question was in any degree contagious before the vessel reached Boa Vista, and we have a right to look for proof both at Sierra Leone and the Gambia, where the evidence is against contagion. At Boa Vista the reverse is the case; for the whole history of the progress of the fever subsequent to the landing of the crew on the small island proves it to have then possessed highly infectious qualities. I would say, then, that the contagious properties which marked the 'Eclair' fever at Boa Vista were *acquired or contingent,* and not primarily or essentially belonging to it." P. 110.

Dr. Mc William, in drawing his observations to a close, alludes to what he considers the injudicious reluctance of Sir William Pym to permit the immediate removal of the sick from the Eclair, upon her arrival at the Motherbank, either on board a hulk of proper size, or to one of the wings of Haslar hospital, "to be appropriated exclusively for them," as was proposed at the time by Dr. (now Sir John) Richardson.

"This humane proposal might in my opinion have been acted upon, without any risk of the public health being endangered; for the history of yellow fever in ultratropical countries shows that a continued high temperature beyond that of the summer heat of England is necessary to keep its poison in a state of activity; and that when it has prevailed at Gibraltar, Cadiz, Malagar, and Leghorn, it has always been preceded by a certain duration of high temperature. The Superintendent-General of Quarantine objected most decidedly to the sick crew being landed at Haslar, on the ground 'of the disastrous consequences to the mercantile interest of this country, in the event of the 'Eclair' being admitted to pratique;' for this reason alone, 'and without entering into a discussion relative to the public health,' did Sir William Pym oppose the removal of the sick crew to Haslar. One would have indeed hardly expected that the Superintendent-General would have much dread of the danger of infection, from the crew of the 'Eclair' being landed in the month of October in the commodious, cool, and well-ventilated wards of the finest hospital in the world, after reading in his work that the contagious powers of yellow fever 'are destroyed by cold, or even by a free circulation of moderately cool air.'" P. 111.

The general Conclusions of Dr. M.'s Report stand thus:—

"First That the fever on board the 'Eclair' was primarily the remittent of the African coast, which is not a contagious disorder, but that the disease acquired contagious qualities in virtue of a series of causes.

"Second, That although there exists on the Island of Boa Vista a physical cause capable of producing remittent fever, yet it does not appear that that cause was in action when fever broke out in September 1845, and that the island was quite healthy when the 'Eclair' arrived there.

"Third, That the disease of which the Portuguese soldiers died at the Fort (Duke of Braganza) on the small island, was that which afterwards ravaged Boa Vista, and the same as that which prevailed among the crew of the 'Eclair.'

"Fourth, That the fever was propagated throughout the island almost exclusively by direct intercourse with the sick, there being only two cases in which there appears any probability of persons having been infected in any other way.

"Fifth, That although those who had passed through the fever were much less liable to the disease than those who had not, yet it would appear that a person having had one attack, possesses no absolute protection against a second attack.

"Sixth, That connecting the whole of the circumstances attending the arrival and stay of the 'Eclair' at Boa Vista with those under which the disease appeared on the small island, and afterwards on Boa Vista itself, leaves no doubt of its having been introduced by the 'Eclair.'

"Seventh, That in all probability the mortality from fever on the island was much increased by the want of proper nourishment for the people, as well as by the total absence of medical assistance for some months.

"Eighth, That the disease had in no case spread to any of the other islands of the Cape de Verd archipelago." P. 112.

In taking leave of Dr. M'William's report, we cannot but express our surprise that Sir William Burnett, in transmitting it to the Admiralty, should still persist in asserting, now that the complete circumstances of the case have been ascertained and made public, that there was no case of the fever at Boa Vista for a month after the departure of the Eclair! Such hardihood of assertion surely savours more of pertinacity than of calm conviction. Sir William Burnett himself, in his former communication upon the subject, (vide the number of the Med. Chir. Rev. for July, 1846, p. 228,) candidly admitted that—" if it can be fully and satisfactorily shewn that any person, who had visited the ship or tents where the sick were placed, contracted the fever in question and communicated it to others, and they to other persons in succession who had never visited the ship or sick, then there can be no reason to doubt the infectious nature of the disease; but if nothing of this kind has taken place, then the conclusion must be that the disease is not infectious, and is therefore incapable of being communicated; in either case settling this long-contested question." Can any unprejudiced reader of Dr. M'William's report hesitate for a moment as to his opinion? We cannot but suppose that Sir William Burnett allowed himself to prejudge the question at first, and that he was afterwards unwilling to recede from the position he had taken up. His stinted and ungracious recommendation of Dr. M.'s report must surely have proceeded from the same feeling of disappointed expectation.

From one Sir William we pass on to another Sir William. He of the Quarantine board is, as might be expected, much more complimentary to Dr. M'William than the *chef* of the Navy Medical Department. The Report is declared by the former to be " a most valuable document," displaying "great judgment, perseverance and impartiality," and its author is said to " deserve well of his country for the manner in which he has executed his mission." Strange!—is it creditable?—that the very same act and work should be so differently regarded by men equally professing the same regard for truth and candour.

As a matter of course, Sir William Pym points to the cases of Georgio the store-keeper, of Luis Pathi, and of the two Portuguese soldiers at the Fort as affording incontrovertible evidence of the introduction of the fever by the 'Eclair,' and of its spreading by infection. We quite agree with him in this respect: the facts are too strong to be gainsaid, and too conclusive to be explained away. But we cannot see how the evidence, brought forward by Dr. M., can anyhow be said to finally settle the question as to malignant Remittent and Yellow Fever being essentially distinct diseases. Dr. McWilliam evidently thinks quite otherwise, as we have already seen. Sir William then enumerates what he deems to be the distinguishing features between the two diseases. He makes the presence of the *black vomit* to be the characteristic or discriminating symptom of the true pestilence, and this symptom he (unguardedly, it must be presumed) says "only shews itself in the persons of natives of a cold climate." He does not hesitate to declare that it is "a native of and peculiar to the West Coast of Africa," and that it "has been at various times imported into different islands and countries; viz. to the different West India islands, the island of Ascension, and the different ports of Spain and North America, as in the year 1845 into the island of Boa Vista." With respect to the assertion that "it has a singular and peculiar character; viz., that, like Small-pox, it attacks the human frame but once," we shall only now say, in addition to what has been suggested in a former page, that the character or attribute in question is by no means so *singular* or *peculiar* as Sir William and some other writers imagine. All fevers of a continued type, (whether originally or only secondarily so,) and more especially when they have once occurred in a severe form, leave a greater or less degree of insusceptibility to a relapse or recurrence. Second attacks of true Typhus are comparatively of very rare occurrence. We have already seen that the same holds true of the Ardent fever, and, we doubt not, of *malignant* Remittent also.

The general assertion, too, of Sir William that the endemic fevers of intertropical countries are not infectious has been shown to be, when taken in an unqualified sense, not strictly correct: they are unquestionably liable, now and then, to become so under circumstances of crowding, want of cleanliness, imperfect ventilation, &c.

With respect to the statement that "it appears evident from the history of the 'Eclair,' that she had both diseases on board at different and distinct periods; viz., the marsh or mild during the months of April, May and June, and the yellow or Bulam fever with black vomit, from the 23rd of July to the time of her arrival in England," we can only say that it is very far from being evident. Dr. Mc William, as we have seen, interprets the matter very differently, and, as it seems to us, in the right way. Sir Wm. indeed, fixes the exact day when the first case in which there was black vomit occurred, viz., on the 27th of July, the fourth day after the steamer left Sierra Leone. But not only is there an utter want of evidence to warrant this statement,* but there is a very strong presumption that this symptom had been observed, in a partial degree at least, at a much earlier period; for (as quoted in our previous article) we read, in the account of three *post-mortem* examinations in the month of April, the following entries made by Dr. Maconchy:—"the stomach contained some reddish glairy matter"—"the stomach was distended with dark serous fluid, mixed with black flakes." What, pray, were those appearances but evidences of black vomit having, either actually or all but, taken place? and when it is mentioned in addition that, "not a clot was seen in the body, but fluid

---

* It is amusing to notice how Dr. Copland, in his narrative of the fever on board, seeks to reconcile Sir William's statement on this point with the lack of any evidence to prove it. While he admits that "no description or details are given in the official papers," he nevertheless tells us that "on this point it is impossible for Sir William to have been mistaken; seeing that his experience of this distemper in the West Indies and in the South of Spain has been greater than that of any other physician whatever." What has his experience to do with the matter?

blood exsuded from the nostrils and blistered back," can we resist the conclusion that already the fever on board the 'Eclair' had begun to exhibit symptoms of incipient malignancy? Moreover, it is left utterly unexplained by Sir William Pym, where his true yellow or Bulam fever was caught. It was not prevailing at Sierra Leone at the time of the departure of the Eclair; and all the other ships of the squadron were healthy at the time, and remained so. Both of these considerations appear to us to render it very much more probable that the already existing fever on board of the steamer *became*, from a variety of circumstances all tending to the deterioration of the health of the crew, infectious and fatally malignant, than that an entirely new disease, *nova pestis*, was introduced on board immediately before the vessel left Sierra Leone.

It is curious to observe how apt a person, who has taken up strongly any favourite hypothesis, is to assimilate, and as it were incorporate, all facts that present themselves with that hypothesis. Sir William Pym, in his zeal to prove the great fatality of the real Yellow fever, and consequently the danger of its being imported into this country, actually quotes from an anonymous newspaper a list of ships on the African coast, with their mortality, during a period of eight months in the year 1837-38. Out of 650 men, no fewer than 308 perished; a truly frightful loss! But how, may we ask, does Sir William know that every one of these fatal cases was his genuine yellow or Bulam fever? Is it not much more probable that a very large proportion arose from that very fever, viz. the endemic remittent, which he so emphatically declares to be essentially distinct from the former, and which we know to be abundantly fatal?

Sir William still adheres to the propriety of the advice he gave upon the arrival of the 'Eclair' in England not to land the sick, as was proposed by Sir J. Richardson, at Haslar Hospital, upon the ground that the landing of them at Boa Vista was the cause of the introduction of the disease into that island:—not a very valid reason, it must be confessed. His allusion too to the disease being arrested at Moradinha, in the beginning of June, by the measures recommended by Dr. Mc William, is surely straining a more than doubtful point to the very uttermost: the fever was all but extinct upon the island at the time!

In concluding our notice of the "Eclair fever," we may remind our readers that although four of the officers of the "Growler," who went on board of the former steamer at Boa Vista, were attacked very soon after, there is no evidence to show that the disease spread to any other of the Growler's crew. But what is very remarkable is that, while this vessel lay at Woolwich, two of her men, engaged in clearing out her hold (which, it is stated, gave out a very offensive smell), were taken ill on the 10th of October—a month after leaving Boa Vista—and both died in hospital with all the characteristic cases of yellow fever: no other case occurred subsequently, either on board the Growler or among any of the attendants in the hospital on shore. Here, therefore, is another instance of the disease appearing to be connected with a foul and unwholesome state of the vessel's hold, and of the danger of men being exposed to the effluvia thence proceeding. It is much to be regretted that Mr. Carter, the surgeon of the Growler, has not given the public any particulars respecting the health of her crew, notwithstanding the appeal that has been made to him.

Already so large an amount of space has been occupied with the preceding details, that we have left ourselves only a few lines to allude to the principles which should guide a medical man in his endeavours to prevent the introduction and arrest the diffusion of the Yellow fever. And here, to prevent all mistake, let it be understood that we are alluding to a fever of hot climates, which, *in whatever manner it may have originated,* is accompanied with symptoms of a dissolved and putrescent state of the fluids, as indicated by the occurrence of black vomit, sanguineous discharges from the bowels and other passages, and by a luridly-yellow discolouration of the surface. That such a disease is liable, under certain favouring circumstances, to exhibit highly infectious properties cannot, we think, be fairly disputed ; and is not this just what we might have *a priori* expected from its very analogy, we might rather say its close alliance, with other forms of malignant putrid fever? Cullen was quite right, we are convinced, in making it one of the several varieties or modifications of Typhus; its peculiar characters being the result of climatorial and other appreciable influences.

It would be easy to point out many features that are common to them all; and we verily believe that it has been from a neglect of this very fact that there has been so much discrepancy of opinion amongst medical men in reference to the one we have been treating of. That petechial typhus is apt to spread by infection will not be denied: and the same thing seems to hold good of malignant yellow fever.* The virulence of the infection, and consequently the risk of propagation, in both are proportionate to the degree of the dissolution and putrescency of the fluids, and the corresponding malignancy and fatality of the disease.

There is but little occasion to entangle ourselves in controversies as to there being any essential difference between one intertropical fever and another, or as to the alleged original *habitat* of a particular species in this part of the world or in that. It is sufficient for the practical physician to recognise the simple and intelligible principle that, whenever a fever in a hot climate begins to exhibit symptoms which clearly indicate that the blood is "touched corruptibly," it is high time for him to have recourse to precautionary measures to mitigate or arrest its diffusion from the sick to the healthy. And what are these measures?—the very same that every prudent man would resort to, in this country, in the case of Typhus. Do not crowd the sick together, but keep them as much apart as possible; maintain the greatest cleanliness and freedom of ventilation in their chambers and abodes; separate the healthy, and prevent all unnecessary intercourse between them and the sick ; remove all causes, as far as may be practicable, of existing insalubrity; be of good courage, be not afraid. It is always useful to have the healthy well-fed and actively occupied. Wherever the sick have been located, the cabin, room, or ward should not be occupied by persons in health until it has undergone a thorough cleansing and purification; and here we cannot but express our opinion that the use of disin-

* Much of what has been said on the Typhoid form of Dysentery, in a previous page of our present number, is strictly applicable to the history of the Yellow fever, and supersedes the necessity of further remark in this place.

fectant gases is not sufficiently appreciated in the present day on board ship, in barracks, and so forth, wherever numbers of the sick are apt to be accumulated.

While recognising the importance, nay the necessity, of such measures as these, we need scarcely say that much of the present system of Quarantine restraint, in reference to yellow fever, &c., is very needlessly vexatious and oppressive. The truth is, that the whole *regime* requires revision and remodelling. It should form a part of a general code of sanitary discipline; and the quarantine board should be but a branch of a general board of health. There has been a vast deal of most exaggerated and foolish alarm about the introduction of foreign pestilential diseases, while, all the time, we have been overlooking the more formidable pestilences that are continually at our very doors.* Much of this extravagant fear has arisen from keeping the subject of quarantine laws and enactments apart and distinct from the consideration of those measures, that should be had recourse to against the diffusion of our own home-bred fevers. The general plan of prevention, in both cases, is the same; nor need there be any great difference in the particular measures to be adopted. To impose a fixed period of 20, 30, or 40 days upon suspected, or even upon infected, vessels, &c., without regard to the particular circumstances of each individual case, is manifestly absurd. No medical man would recommend such a practice in reference to the worst forms of European petechial fever.

As to the yellow fever being ever imported by the *sound* cargoes of vessels coming from infected ports, there is scarcely an atom of evidence to shew that such has ever been the case; and, even with respect to the bed or body-clothes of passengers, the risk of its introduction in this way seems to be indeed but very small. Nevertheless it will always be prudent to avoid this risk, especially as it can be so very easily done with little trouble or expense.

Much more attention ought to be paid to the condition of ships' holds than is usually the case. That a foul hold is a not unfrequent cause of bad fever on board, a fever that may eventually acquire an infectious character, is much more than probable. If masters and owners knew that the duration of their quarantine would in a material degree depend upon the state of their vessels, a great deal of the expense and delay to which they are at present subjected might, with perfect safety to the public, be avoided.

---

* How strikingly is this exemplified in the New York Report. The number of deaths, in the Marine hospital on the quarantine ground, is ten-fold and upwards greater from small-pox than it usually is from yellow fever; and yet, during most of the year, there is no quarantine whatsoever against the former disease.

